# COVID-19 vaccination coverage for half a million non-EU migrants and refugees in England

**DOI:** 10.1038/s41562-023-01768-6

**Published:** 2023-12-04

**Authors:** Rachel Burns, Sacha Wyke, Max T. Eyre, Yamina Boukari, Tina B. Sørensen, Camille Tsang, Colin N. J. Campbell, Sarah Beale, Dominik Zenner, Sally Hargreaves, Ines Campos-Matos, Katie Harron, Robert W. Aldridge

**Affiliations:** 1 Centre for Public Health Data Science, Institute of Health Informatics, University College London, London, UK; 2 Department of Health and Social Care, Office for Health Improvement and Disparities, London, UK; 3 Department of Disease Control, Faculty of Infectious and Tropical Diseases, London School of Hygiene and Tropical Medicine, London, UK; 4 Immunisation and Vaccine Preventable Diseases, UK Health Security Agency, London, UK; 5 Institute of Epidemiology and Health Care, University College London, London, UK; 6 Global Public Health Unit, Wolfson Institute of Population Health, Barts and The London School of Medicine and Dentistry, Queen Mary University of London, London, UK; 7 Infection and Population Health Department, Institute of Global Health, University College London, London, UK; 8 Institute for Infection and Immunity, St George’s University of London, Cranmer Terrace London, London, UK; 9 UK Health Security Agency, London, UK; 10 UCL Great Ormond Street, Institute of Child Health, University College London, London, UK

**Keywords:** Epidemiology, Health services

## Abstract

Despite evidence suggesting that some migrants are at risk of under-immunization and have experienced severe health inequities during the pandemic, data are limited on migrants’ COVID-19 vaccine coverage globally. Here we linked data from non-European Union migrants and resettled refugees to the national COVID-19 vaccination dataset in England. We estimated patterns in second and third dose delays and overdue doses between 12 December 2020 and 20 April 2022 by age, visa type and ethnicity. Of the 465,470 linked records, 91.8% (427,073/465,470) of migrants received a second dose and 51.3% (238,721/465,470) received a third. Refugees had the highest risk of delayed second (adjusted odds ratio 1.66; 95% confidence interval 1.55–1.79) and third dose (1.55; 1.43–1.69). Black migrants were twice as likely to have a second dose delayed (2.37; 2.23–2.54) than white migrants, but this trend reversed for the third dose. Older migrants (>65 years) were four times less likely to have received their second or third dose compared with the general population in England aged >65 or older. Policymakers, researchers and practitioners should work to understand and address personal and structural barriers to vaccination for diverse migrant populations.

## Abstract (web summary)

The authors linked data from non-European Union migrants and resettled refugees to the national COVID-19 vaccination dataset in England, demonstrating disparities in vaccination timing and coverage.

## Main

The coronavirus disease 2019 (COVID-19) pandemic disproportionately impacted some migrant communities in high-income countries such as the United Kingdom, placing them at higher risk of contracting severe acute respiratory syndrome coronavirus 2 and experiencing severe disease and death from COVID-19 (ref. ^1^). Here migrants are defined as people born outside of the United Kingdom, including those who choose to migrate (for example, work, study or join families) and those who may have been forced to migrate (for example, refugees and asylum seekers). Migrants had greater exposure to COVID-19 due to a range of risk factors, such as working in public-facing occupations, living in high-density accommodation or large, multi-generational households and facing barriers to accessing healthcare and public health messaging^2,3^. In 2020, all-cause mortality increased substantially more among people who migrated to the United Kingdom from several global regions than among people who were born in the United Kingdom^4^. Moreover, migrant populations on the whole are known to be an under-immunized group in Europe for routine vaccines^5,6^, and evidence of low COVID-19 vaccine uptake in some migrant groups is emerging from several European countries^7–10^.

Recognizing these risks, high COVID-19 vaccination uptake in migrant populations is important. Although COVID-19 vaccines were freely available to all irrespective of immigration status in the United Kingdom, research has shown low vaccine intent among some migrant groups^11–13^. The literature identifies the following barriers to COVID-19 vaccination among migrant populations: lack of trust in the government and health system, limited culturally sensitive and language-appropriate messaging, and logistical challenges to accessing vaccination services^11–13^. These barriers to uptake can vary depending on an individual’s migration status (for example, visa type), which can reveal information about migration drivers, broader socio-economic determinants of health, and access to public services. Routine data collected on vaccine uptake in the United Kingdom has focused on ethnicity, with several ethnic minority groups (containing both United Kingdom born and non-United Kingdom born) reporting low vaccination uptake^14^. A study in England, which used the 2011 Census to identify country of birth in national COVID-19 vaccination data, found that migrants from Black African, South Asian and Other ethnicities had a higher first dose uptake than their United Kingdom-born counterparts, but this study excluded recent migrants (arriving after 2011) and lacked information on visa type^15^. The COVID-19 Health Inequalities Monitoring for England (CHIME) tool monitors COVID-19 vaccination in migrants but does not include information about visa type or ethnicity^16^.

Most reports on COVID-19 vaccination coverage in migrant populations rely on qualitative assessments or indirectly estimate potential uptake through vaccine intention or acceptability. So far, there have been limited large-scale population-based studies on COVID-19 vaccination coverage in migrant populations in the United Kingdom probably due to challenges associated with migrant identification in national immunization datasets. The Million Migrant cohort, which consists of over 2 million non-European Union (EU) migrants and resettled refugees who migrated to the United Kingdom between 2005 and 2020, provides a unique opportunity to identify and describe migrants in UK routine COVID-19 immunization records^17^. Understanding differences in vaccine coverage and uptake is essential to informing COVID-19 vaccine roll out in the immediate term, future migrant-inclusive pandemic preparedness plans and service planning for routine immunization programmes.

In this Article, we aimed to describe the trends and variation in second and third COVID-19 vaccine delays and overdue or missed doses across different migrant subgroups compared with the general population in England. The objectives were:

1. To investigate the age-specific proportions and odds of having a delayed second or third dose between migrant subgroups by visa type and ethnicity,
2. To investigate the age-specific proportions of an overdue second or third dose by the end of the study period for migrant subgroups by visa type and ethnicity compared with the general population in England.

## Results

### Cohort characteristics

Of the 1,674,268 individuals aged ≥16 years within the Million Migrant cohort, 1,045,786 (62.5%) linked to a National Health Service (NHS) number in Personal Demographic Service (PDS). Of these, 465,470 (44.5%) were linked by NHS number to at least one National Immunisation Management Service (NIMS) COVID-19 vaccination record as of 20 April 2022 and were included in the Million Migrant-NIMS cohort (Appendix G for data flow diagram and linkage results). Relative to the Million Migrant cohort with NHS numbers, the Million Migrant-NIMS cohort had fewer individuals from East Asia and Pacific (20.1% in Million Migrant-NIMS versus 36.0% Million Migrant cohort with NHS numbers) and more from South Asia (53.1% versus 41.2%) and had fewer individuals on student visas (24.2% versus 43.7%) and more on settlement and dependent visas (44.0% versus 26.4%) (Table 1 and Supplementary Table 7 in Appendix G).

**Table 1:** Demographic characteristics of the total Million Migrant cohort, the Million Migrant cohort with an NHS number, and those within the Million Migrant-NIMS cohort who had at least one COVID-19 vaccination as of 20 April 2022

	Million Migrant cohort *N* = 1,674,268 (100.0%)	Million Migrant cohort with an NHS number *N* = 1,045,786 (62.5%)	Million Migrant-NIMS cohort^a^ *N* = 465,470 (27.8%)
Sex			
Female	826,455 (49.4%)	557,900 (53.3%)	260,524 (56.0%)
Male	847,813 (50.6%)	487,886 (46.7%)	204,946 (44.0%)
Age* (years)			
16–17	20,262 (1.2%)	14,192 (1.4%)	3,688 (0.8%)
18–29	668,838 (39.9%)	484,651 (46.3%)	159,899 (34.4%)
30–39	651,314 (38.9%)	383,483 (36.7%)	204,899 (44.0%)
40–49	228,433 (13.6%)	119,222 (11.4%)	69,767 (15.0%)
50–54	43,599 (2.6%)	18,539 (1.8%)	11,410 (2.5%)
55–59	27,602 (1.6%)	9,667 (0.9%)	5,881 (1.3%)
60–64	13,910 (0.8%)	5,578 (0.5%)	3,452 (0.7%)
65–69	7,256 (0.4%)	3,632 (0.3%)	2,358 (0.5%)
70–79	9,605 (0.6%)	5,051 (0.5%)	3,271 (0.7%)
80+	3,449 (0.2%)	1,771 (0.2%)	845 (0.2%)
Region of origin			
East Asia and Pacific	565,949 (33.8%)	376,825 (36.0%)	93,583 (20.1%)
Europe and Central Asia	43,955 (2.6%)	32,672 (3.1%)	12,559 (2.7%)
Latin America and Caribbean	889 (0.1%)	569 (0.1%)	227 (0.0%)
Middle East and North Africa	32,000 (1.9%)	19,029 (1.8%)	11,301 (2.4%)
North America	307 (0.0%)	255 (0.0%)	159 (0.0%)
South Asia	741,298 (44.3%)	430,558 (41.2%)	247,141 (53.1%)
Sub-Saharan Africa	289,832 (17.3%)	185,862 (17.8%)	100,488 (21.6%)
Missing	38 (0.0%)	16 (0.0%)	12 (0.0%)
Entry visa type			
Students	812,186 (48.5%)	457,170 (43.7%)	112,626 (24.2%)
Work	190,596 (11.4%)	125,808 (12.0%)	67,198 (14.4%)
Settlement and dependents	397,169 (23.7%)	276,338 (26.4%)	204,937 (44.0%)
Family reunion	76,386 (4.6%)	51,173 (4.9%)	27,468 (5.9%)
Refugee	20,838 (1.2%)	12,151 (1.2%)	8,310 (1.8%)
Other	161,780 (9.7%)	117,096 (11.2%)	41,881 (9.0%)
Missing	15,313 (0.9%)	6,050 (0.6%)	3,050 (0.7%)
Length of time in United Kingdom^b^ (years)			
<2	352,661 (21.1%)	270,796 (25.9%)	101,158 (21.7%)
2–3	360,476 (21.5%)	278,209 (26.6%)	129,314 (27.8%)
4–5	281,023 (16.8%)	201,179 (19.2%)	73,375 (15.8%)
6–7	205,957 (12.3%)	76,099 (7.3%)	43,040 (9.2%)
>8	474,151 (28.3%)	219,503 (21.0%)	118,583 (25.5%)
Region of England^c^			
East Midlands	56,475 (3.4%)	56,475 (5.4%)	25,328 (5.4%)
East of England	71,533 (4.3%)	71,533 (6.8%)	40,564 (8.7%)
London	272,338 (16.3%)	272,338 (26.0%)	143,529 (30.8%)
North East	33,191 (2.0%)	33,191 (3.2%)	14,146 (3.0%)
North West	94,966 (5.7%)	94,966 (9.1%)	48,795 (10.5%)
South East	126,097 (7.5%)	126,097 (12.1%)	68,828 (14.8%)
South West	48,618 (2.9%)	48,618 (4.6%)	22,118 (4.8%)
West Midlands	94,760 (5.7%)	94,760 (9.1%)	48,022 (10.3%)
Yorkshire and the Humber	83,303 (5.0%)	83,303 (8.0%)	36,864 (7.9%)
Other	24,799 (1.5%)	24,799 (2.4%)	1279 (0.3%)
Missing	768,188 (45.9%)	139,706 (13.4%)	15,997 (3.4%)
Ethnicity^d^			
Black	–	–	56,664 (12.2%)
Mixed	–	–	14,338 (3.1%)
Other	–	–	114,950 (24.7%)
South Asian	–	–	179,460 (38.6%)
Unknown	–	–	74,597 (16.0%)
White	–	–	25,461 (5.5%)
COVID-19 dose			
First	–	–	465,470 (100.0%)
Second	–	–	427,073 (91.8%)
Third	–	–	238,721 (51.3%)

^a^Million Migrant-NIMS cohort only includes those with match rank 1–5.

^b^Age and length of time in the United Kingdom as of 12 December 2020 for Million Migrant cohort and Million Migrant cohort with an NHS number and as of first COVID-19 vaccine dose for Million Migrant-NIMS cohort.

^c^Other within ‘Region of England’ includes Crown Dependencies, Scotland, Northern Ireland and Wales.

^d^Ethnicity available only for those who linked to the Million Migrant-NIMS cohort dataset.

### Delayed vaccination

**Fig. 1:**
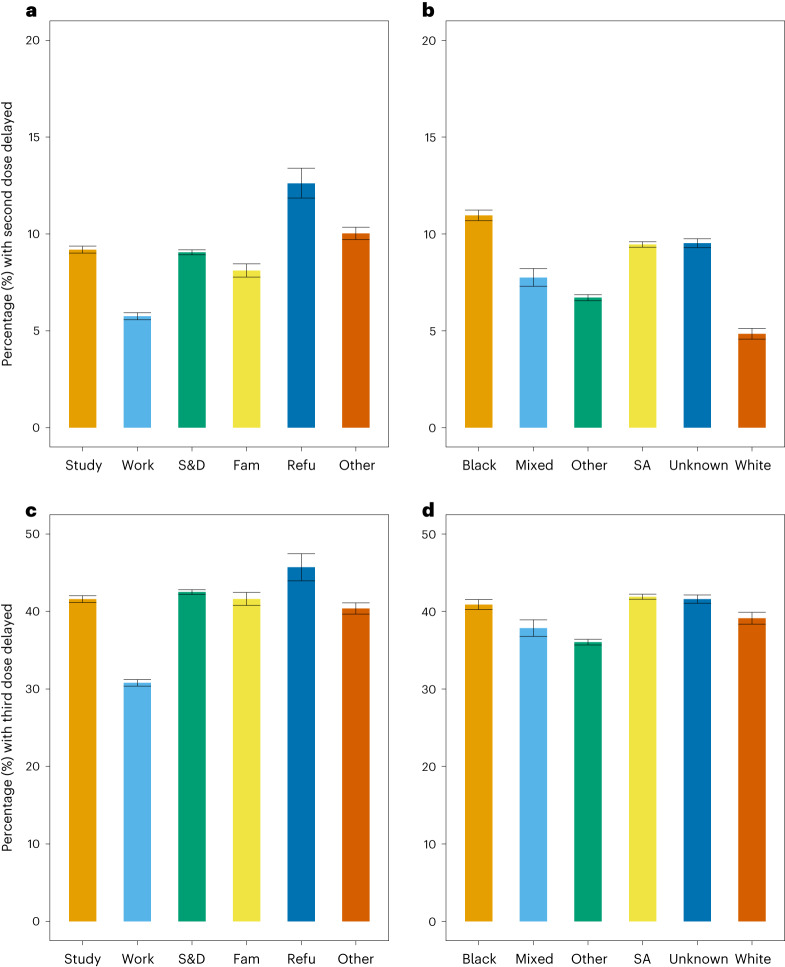
Delayed second and third COVID-19 dose vaccinations by visa type and ethnicity. **a**–**d**, Proportion (%) and error bars represent 95% CI of individuals over 18 years old with second dose delayed by visa type (**a**), second dose delayed by ethnicity (**b**), third dose delayed by ethnicity (**c**), and third dose delayed by ethnicity within the Million Migrant-NIMS cohort as of 20 April 2022 (**d**). Note: different *y*-axis limits for second dose delayed (**a** and **b**) and third dose delayed (**c** and **d**). For sample sizes, see Supplementary Table 8. Study, students; Work, workers; S&D, settlement and dependents; Fam, family; Refu, refugee; SA, South Asian.

Compared with other visa types, refugees had the highest proportion of delayed second and third doses, with 12.6% (95% confidence interval (CI) 11.8–13.4%) second doses delayed and 44.4% (95% CI 42.7–46.1%) third doses delayed (Fig. 1a,c). Conversely, individuals on work visas were least likely to be delayed for either dose, with 5.7% (95% CI 5.6–5.9%) second doses delayed and 25.9% (95% CI 25.5–26.3%) third doses delayed. Similar trends were seen when stratifying by age group (Supplementary Figs. 3 and 4 in Appendix H). Migrants with a white ethnicity were half as likely to be delayed for their second dose (4.8%; 95 CI 4.6–5.1%) as migrants with a Black ethnicity (11.0%; 95% CI 10.7–11.3%). These differences among ethnic groups were lost for the third dose (Fig. 1b,d; see age group stratification in Supplementary Figs. 5 and 6 in Appendix H).

In the multivariable regression analysis (Table 2), refugees had significantly higher odds of receiving a delayed second dose relative to the baseline group of settlement and dependent visa (adjusted odds ratio (aOR) 1.66; 95% CI 1.55–1.79). This was also the case for third doses (aOR 1.55; 95% CI 1.43–1.69). Individuals on work visas were half as likely to receive a delayed second dose (aOR 0.62; 95% CI 0.60–0.75) or a delayed third dose (aOR 0.64; 95% CI 0.61–0.66) than those on other visas. Migrants in all ethnic minority groups had a significantly higher adjusted odds of having a delayed second dose compared with migrants in the baseline White group. Individuals in the Black group were more than two times more likely to have a delayed second dose (aOR 2.37; 95% CI 2.23–2·54), followed by individuals in the South Asian group (aOR 1.99; 95% CI 1.87–2.12) relative to the White group. Different trends were observed for the third dose, with individuals within the Mixed, Other and South Asian groups having a lower odds of delay than the White group.

**Table 2:** Multivariable logistic regression model showing aORs and 95% CI for second and third COVID-19 dose delayed by ethnicity and visa type

	Second dose delayed (*N* = 424,146)				Third dose delayed (*N* = 229,837)			
	Total	aOR	95% CI	*P* value^*^	Total	aOR	95% CI	*P* value
Visa type**								
Settlement	190,774	—	—	—	37,976	—	—	—
Other	34,924	0.99	0.95, 1.03	0.6	11,667	1.04	0.99, 1.09	0.14
Refugee	7,217	1.66	1.55, 1.79	<0.001	3,060	1.55	1.43, 1.69	<0.001
Family reunion	24,604	0.88	0.84, 0.92	<0.001	5,754	1.3	1.22, 1.38	<0.001
Students	99,137	0.87	0.84, 0.89	<0.001	25,196	1.09	1.05, 1.13	<0.001
Work	64,729	0.62	0.60, 0.65	<0.001	27,322	0.64	0.61, 0.66	<0.001
Ethnicity^***^								
White	24,112	—	—	—	14,290	—	—	—
Black	51,321	2.37	2.23, 2.54	<0.001	22,380	0.98	0.94, 1.03	0.4
Mixed	13,299	1.64	1.50, 1.78	<0.001	6,378	0.85	0.79, 0.90	<0.001
Other	103,461	1.32	1.24, 1.41	<0.001	46,120	0.76	0.73, 0.80	<0.001
South Asian	168,342	1.99	1.87, 2.12	<0.001	2,499	0.75	0.69, 0.83	<0.001
Unknown	63,611	1.84	1.73, 1.97	<0.001	19,308	1.03	0.98, 1.08	0.2

^*^P values calculated on the basis of two-sided Wald test following standard generalized linear model methodology

**Visa type controlling for age, sex and ethnicity;

***Ethnicity controlling for age and sex. Note: Individuals under 18 and/or without a second dose were excluded from second dose delayed (*N* = 43,758) and individuals under 18 and/or without a third dose were excluded from third dose delayed (*N* = 238,067).

### Overdue vaccination

**Fig. 2:**
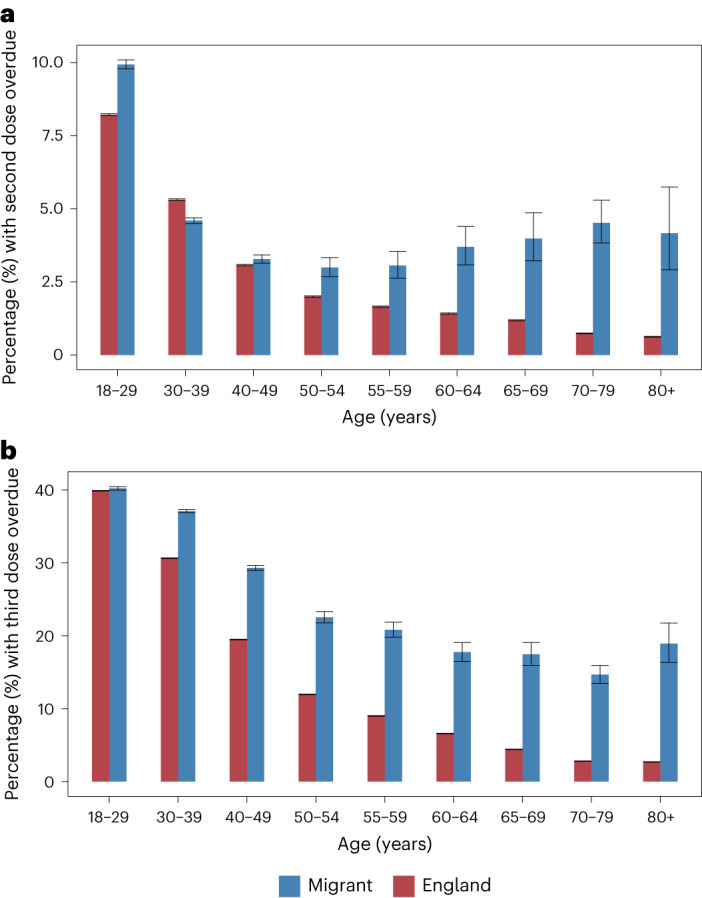
Overdue second and third COVID-19 dose vaccinations for migrants and the general population in England. **a**,**b**, Proportion (%) and error bars represent 95% CI of individuals by age group with second dose overdue (**a**) and third dose missing (**b**) comparing the Million Migrant-NIMS cohort (blue) and the England cohort (red) as of 20 April 2022. For sample sizes, see Supplementary Table 9.

Migrants were more likely to be overdue for their second dose than the England cohort in all age categories except 30–39 (Fig. 2a). This trend increased with age, with migrants aged ≥65 almost four times more likely to not have received their second dose than their English counterparts (4.0%; 95% CI 3.3–4.9% in migrants aged 65–69 compared with 1.19%; 95% CI 1.17–1.21% in England). Similarly, migrants were more likely to be overdue their third dose than the England cohort in all age groups, with the largest differences for older ages (Fig. 2b). Migrants aged ≥65 were at least four times more likely to be overdue their third dose than the England cohort (17.5%; 95% CI 16.0–19.1% in migrants aged 65–69 compared with 4.44%; 95% CI 4.39–4.47% in England). A similar pattern in overdue second doses was retained in the sensitivity analyses when both the study follow-up period was shortened (from 20 April 2022 to 1 June 2021) and newly arrived, shorter-term migrants were excluded to estimate travel out of England (Supplementary Figs. 7 and 8 in Appendix I).

**Fig. 3:**
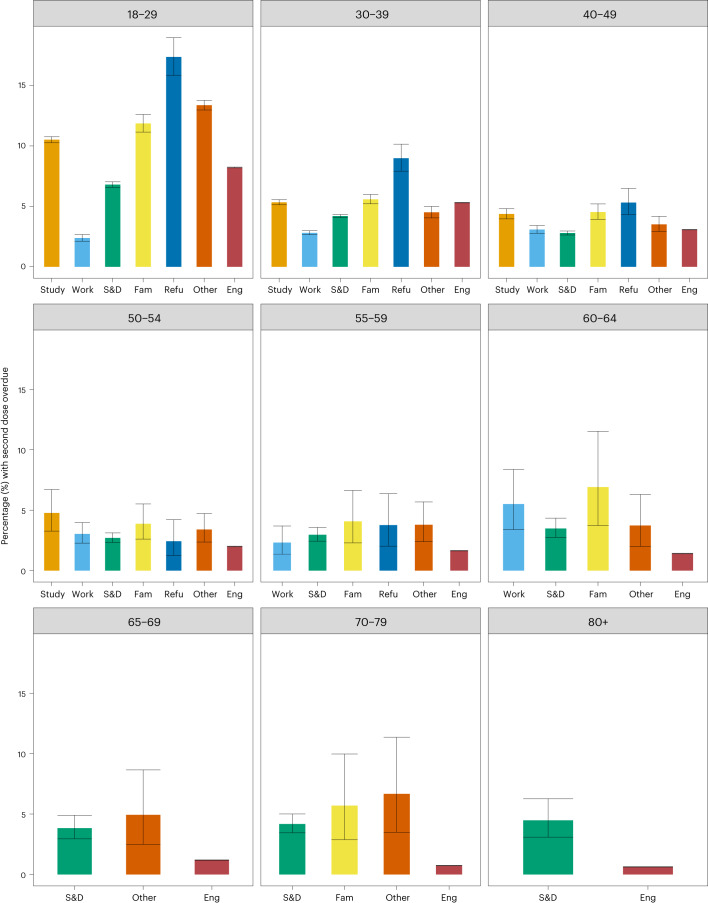
Overdue second COVID-19 dose vaccinations by visa type and age. Proportion (%) and error bars represent 95% CI of individuals by age group with second dose overdue comparing visa type and England cohort as of 20 April 2022. For sample sizes, see Supplementary Table 10. Study, students; S&D, settlement and dependents; Fam, family; Refu, refugee; Eng, England.

Differences in the proportion of second and third doses overdue emerged by visa type (Fig. 3 and Supplementary Fig. 9 in Appendix I). Younger refugees (age 18–39) had the highest proportion of overdue second doses than other visa type, with 17.4% (95% CI 15.9–19.0%) in refugees aged 18–29 and 9.0% (95% CI 7.9–10.1%) in refugees aged 30–39 overdue compared with 8.21% (95% CI 8.18–8.25%) and 5.31% (95% CI 5.28–5.34%) in England, respectively (Fig. 3). This trend persisted for third dose overdue with refugees in almost all age categories having the highest proportion of people with a third dose overdue (Supplementary Fig. 9). Migrants on settlement and dependent and family reunion visas had higher proportions of third dose overdue than England across all age groups, with the gap widening significantly for those aged ≥50.

**Fig. 4:**
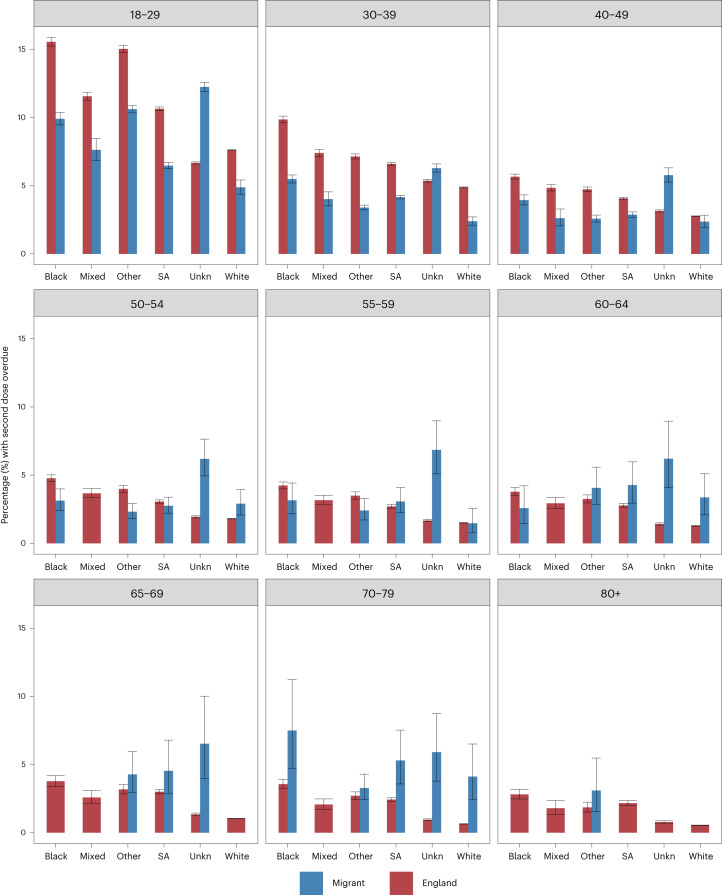
Overdue second COVID-19 dose vaccinations by ethnicity and age. Proportion (%) and error bars represent 95% CI of individuals by age group with second dose overdue comparing ethnicity and England cohort as of 20 April 2022. For sample sizes, see Supplementary Table 11. SA, South Asian; Unkn, Unknown.

Migrants aged under 60 across all ethnic groups except those with an unknown ethnicity had a lower proportion of overdue second doses than their counterparts in England (Fig. 4). However, older migrants (aged over 60) in the Black, South Asian, Other and White ethnic groups had a higher proportion of overdue second doses. A similar pattern was seen for third dose overdue (Supplementary Fig. 10). Older Black migrants (aged over 65) were twice as likely to be missing a third dose than the Black ethnic group in the England cohort, with 32.9 (95% CI 27.4–38.7) and 15.1 (95% CI 14.3–15.6) missing a third dose, respectively.

## Discussion

We present findings on COVID-19 vaccination delays and overdue or missed doses for almost half a million non-EU migrants and resettled refugees compared with the general population in England between 8 December 2020 and 20 April 2022. Refugees and migrants with non-white ethnicity were more likely to have a delayed second or third COVID-19 dose. Refugees and older migrants were more likely to not have received a second or third dose. These data hold immediate relevance to strengthening COVID-19 vaccination for migrants and identify important variability in uptake by age, visa type, and ethnicity.

Our findings highlight more overdue second and third doses (that is, not receiving a subsequent dose) in older migrants compared with their counterparts in England. Slower uptake could have been driven by greater language barriers, limited health literacy, digital exclusion or fear of side effects in older migrants^18–20^. Similar findings were found in a large cross-sectional study in Canada^21^. Decisions to not receive a second or third dose could also be associated with differences in perceived vulnerability to severe COVID-19 outcomes due to lower levels of underlying health issues in migrants compared with the general population in England^22^. Patterns in greater overdue second and third doses for older migrants remained even after the follow-up period was shortened to account for potential emigration or embarkations out of England once international travel resumed. In-depth qualitative exploration on the reasons behind older migrants’ lower uptake is needed especially given the importance of subsequent doses for protection against new variants.

Individuals on work visas were less likely to be overdue for a second or third dose than the England cohort. This may be due to firstly significant proportions of migrants working in health and social care^23,24^ who were initially prioritized for vaccination and eventually included in the United Kingdom government enforced vaccine mandate, and secondly, more stringent work visa sponsorship requirements may favour the entry of migrants with higher socio-economic status, which has been associated with lower vaccine hesitancy^25,26^. Conversely, refugees were more likely to be overdue for both second and third doses than the England cohort and were almost two times more likely to be delayed for their second or third dose, which is consistent with studies on low vaccine intent and under-immunization in other forced migrants^12,27^. Reasons for delays include access barriers, lack of accessible information in appropriate language, fear of vaccine side effects, or lack of familiarity/trust in the health system^12,13,28^. However, these estimates are probably an underestimation of true inequalities among other forced migrants as the refugee participants in this study are resettled refugees who received government support to facilitate early integration with appropriate health and social care services prior to their arrival.

Migrants with non-white ethnicities were more likely to be delayed for their second dose than migrants with a white ethnicity. This could reflect the unique challenges that being both a migrant and an ethnic minority have on vaccine access as a result of healthcare entitlement, language, literacy and other communication barriers^2,29^. As some ethnic minority communities experienced higher severe acute respiratory syndrome coronavirus 2 exposure and subsequent COVID-19 infection, second dose delays could have also influenced by following official guidance to wait at least 4 weeks after an infection before receiving the next dose^30,31^. However, those differences disappeared for the third dose, perhaps due to the rapid roll-out of the booster (third dose) programme or more targeted vaccination campaigns. With evidence clearly demonstrating the disproportionate impact of COVID-19 on ethnic minority groups in England, there was a commitment from the United Kingdom government to support bespoke vaccination campaigns targeting ethnic minority communities to increase vaccine and booster uptake^32^. Still older migrants across all ethnic groups were less likely to return for their third dose than their counterparts within the same ethnic group in the England cohort. Conversely, another study found migrants arriving before 2011 from Black African, South Asian and Other ethnicities had a higher total first dose uptake than their United Kingdom-born counterparts^15^. Further research is needed in exploring predictors of vaccine uptake such as migration status (for example, migrants and non-migrants), visa type within ethnic groups, and socio-economic status.

Strengths of this study include a large study population with information on migration history linked to vaccination records that cover the primary course of COVID-19 vaccination and the initial booster (third dose) campaign recommended for adults in England. Our comparison dataset OpenSAFELY has been found to be largely representative of the general population in England across age, sex, deprivation level, and region^33^.

Key limitations of this study include that the Million Migrant-NIMS cohort is not representative of the entire migrant population in England, with a study population consisting of resettled refugees and migrants from non-EU countries who entered on longer-term visas and have an NHS number. Irregular migrants (for example, undocumented migrants, refused asylum seekers, visa overstayers, and children born to irregular migrant couples), migrants on a temporary visa, EU and European Economic Area migrants, non-EU migrants from low-incidence tuberculosis (TB) countries who do not require a pre-entry TB screening as part of visa application and non-EU migrants who emigrated before the start of either health screening programme were not captured. Importantly, some of these groups like irregular migrants could be in more vulnerable situations. Although only half of the Million Migrant cohort with NHS numbers linked to at least one NIMS COVID-19 record, the demographic profile between the two cohorts was broadly similar but the representativeness of our findings as a result could be limited.

There are several potential sources of bias in the linkage methodology that could impact the generalizability of our findings. An individual might not have linked in either the PDS or NIMS COVID-19 vaccination dataset if they never arrived in England, were resident in Scotland, Northern Ireland or Wales, they were never allocated an NHS number, or their linkage variables were recorded incorrectly or inconsistently. Linkage error due to missing or mis-recorded identifiers could result in a selection bias if the missed matches were not missing completely at random. Because the linkage to a NIMS COVID-19 vaccination record relied on having an NHS number, the cohort excluded migrants without any previous contact with health services and who may have been less likely to receive a vaccine. This selection criteria into the cohort probably overestimated vaccine coverage.

Although there is some certainty that individuals who receive an entry visa to the United Kingdom migrate, when and whether they leave after their visa expires is less certain^34^. Similarly for those with overdue or missed doses, lack of data on emigration during the study period could have led to an over-ascertainment of vaccination overdue. However, for individuals who were most likely to have remained in England for the duration of the study period such as those on refugee, settlement and dependent, and family visas, these estimates are broadly robust and can be helpful indicators of second and third dose uptake^35^. Importantly, the highest rate of vaccination overdue were found in these subgroups and older migrants, even after the study period was shortened and newly arrived migrants on short-term visas (for example, individuals on student, work, and working holiday visas arriving in the last 5 years) were excluded to account for travel out of England.

Several determinants for COVID-19 vaccination coverage were included in this analysis, but no data were available on clinical vulnerability, accommodation (for example, living with someone with a clinical vulnerability or in a care home) or high-risk occupations, all of which were prioritized risk factors for early vaccination in England^36^. We had no information on death, contraindications, or emigration out of the country; all of which could artificially inflate our denominator for vaccination overdue. Our sensitivity analyses measuring the impact emigration (restricting the follow-up period and excluding shorter-term visa holders) showed minimal effect on our estimates. Lastly, we restricted our analyses to those over the age of 16 for first dose and over 18 for second and third doses, limiting the generalizability of our data to those under the age of 16.

Our findings hold immediate relevance to strengthening COVID-19 vaccination and other routine immunizations for migrants and identify important variability in uptake by age, visa type and ethnicity. Most migrants in our cohort, in particular older migrants and refugees, were more likely to be overdue for their second and third doses than England’s general population. These findings highlight slower vaccination uptake for some migrant groups and reinforce the importance of migrant-inclusive policies and services to ensure equitable access^36^. Box 1 summarizes key policy and practice areas of relevance to improve COVID-19 vaccination uptake in migrants in the United Kingdom and other European countries.

It remains important to better understand the drivers of low and delayed vaccine uptake in migrant populations and why refugees and older migrants are not returning to receive their second or third dose of the COVID-19 vaccination. The extent to which these are structural or personal barriers, the role of vaccine hesitancy and misinformation, and the impact of policies resulting in the exclusion of some migrant groups from accessing health and vaccination systems need to be further elucidated. As immunity wanes and new COVID-19 boosters are needed for emerging variants, understanding vaccination coverage for high-risk groups such as migrants will be essential for an adequate and equitable response.

Box 1 Key policy and practice areas requiring action to improve COVID-19 vaccination coverage in migrants Key policy and practice areas requiring action:

1. Co-design context and culturally appropriate vaccination campaigns and research with international, national, regional and local migrant community organizations to ensure accessibility and culturally appropriate services and to better understand barriers and facilitators to vaccination systems on arrival.
2. Explore opportunities with stakeholders to strengthen data collection around vaccination uptake and country of birth, visa category and time since arrival in the host country.
3. Improved consideration of migrant populations in the evaluation and delivery of vaccination programmes for COVID-19 and routine vaccinations.
4. Further research the causes of uptake variations, including differences between different types of migrants.

## Methods

### Study design

We conducted a retrospective population-based cohort study using the Million Migrant cohort linked to England’s NIMS which acts as a national vaccine register for COVID-19 vaccinations. The Million Migrant-NIMS cohort was compared to England’s general population using the publicly available OpenSAFELY dataset^37^. Our study period began on 8 December 2020, the start of the staggered roll-out of the UK national COVID-19 vaccination campaign, and ended 20 April 2022.

All adults aged ≥18 in England were eligible for a primary course of two doses from 18 June 2021. A booster programme (referred to as the third dose) was introduced on 19 September 2021 and was available for everyone aged ≥18 from the end of November 2021 (for more details on the UK COVID-19 vaccination programme, see Appendix A).

### The Million Migrant-NIMS cohort

The Million Migrant cohort consists of two data sources: first, the non-EU migrant pre-entry TB screening dataset collected as part of the UK visa application process, and second, the refugee pre-arrival health assessment dataset collected for all refugees enrolled in a UK refugee resettlement programme (details in Appendix B). The most recent record for individuals aged ≥16 was deterministically linked by forename, surname, date of birth, and sex to the NHS PDS by NHS Digital’s Demographic Batch Service to obtain an individual’s unique patient identifier, NHS number and UK postcode. The Million Migrant cohort (supplemented with NHS numbers from PDS) was deterministically linked by NHS number, date of birth, sex and, where available, UK postcode to the NIMS COVID-19 vaccination dataset held at the United Kingdom Health Security Agency (UKHSA). The linkage followed a stepwise deterministic matching procedure adapted from methodology used by NHS Digital (details on linkage methodology in Appendix C).

### The England cohort

To provide a representative comparison population for England (referred to as the England cohort), we used publicly available aggregate data from the OpenSAFELY cohort^33,37^. The OpenSAFELY cohort included all patients registered with a general practice using either EMIS or TPP software in England. Information on vaccination status was linked back to the participants’ primary care records following their vaccination. Vaccination status and date of vaccination was ascertained by the presence of any recorded COVID-19 vaccine administration code in their primary care record. Total aggregate second and third dose data were available for people aged ≥18 (further details on study data sources in Appendix D).

### Outcomes

Two main outcomes were explored in this analysis: (1) delayed second and third COVID-19 doses and (2) overdue second and third COVID-19 doses.

Delayed vaccination was a binary variable used to compare the age-specific proportions of individuals who had a delayed second or third dose in the Million Migrant-NIMS cohort. Individuals aged >=18 who had their last recorded second or third COVID-19 vaccine dose at least 14 weeks before the end of the study period (20 April 2022) were included. Second doses were considered delayed if they were not received within 14 weeks after the first dose. Third doses were considered delayed if they were not received within 30 days following each age group’s eligibility date as set out by the UK government’s Joint Committee on Vaccination and Immunisation (JCVI). Please note that intervals between doses changed over time as the pandemic evolved (Appendix D).

Overdue or missed vaccination (that is, not receiving a subsequent dose) was a binary variable used to compare the age-specific proportion of individuals aged >=18 who had not received their second or third dose in the Million Migrants-NIMS cohort and England cohort by the end of the study period, 20 April 2022. Individuals were included if they had received their last recorded dose at least 14 weeks before the end of the study (for example, first dose recorded for second dose overdue and second dose recorded for third dose overdue). More detailed outcome definitions and further explanation of date specifications are available in Appendix D.

### Variables

We used the following variables to compare the Million Migrant-NIMS cohort with the England cohort: age, visa type and ethnicity. Age in years was aggregated to match OpenSAFELY age groups: 16–17, 18–29, 30–39, 40–49, 50–54, 55–59, 60–64, 65–69, 70–79 and 80+. Visa type consisted of seven categories: Family Reunion, Settlement and Dependents, Student, Refugee, Work, Other, and England cohort (that is, individuals in England comparison population). Ethnicity was aggregated according to ONS census 2011 categories: White, South Asian, Black, Mixed, Other and Unknown. Ethnicity data were available only for people aged ≥18 in the England cohort. We also selected the following variables to display using descriptive statistics given their association with migration and vaccination uptake: sex, region of origin, year of arrival to England, and region resident in England. No information on death, contraindications or emigration out of England were available. Variable definitions and further description of confounder selection for the final statistical models are available in Appendix D and E.

### Statistical analysis

For the Million Migrant-NIMS cohort, we estimated the proportion of individuals with delayed vaccinations for the second and third doses by age, visa type and ethnicity. We used multivariable logistic regression to model the association between each delayed dose and visa type and ethnicity (for theoretical diagrams and confounder selection, see Appendix E). We estimated the proportion of individuals with overdue vaccinations for second and third dose by age, visa type, and ethnicity for both the Million Migrant-NIMS cohort and the England cohort. Since data on embarkations from England were not available, we restricted the study follow-up period (ending 1 June 2021) and removed newly arrived individuals with shorter-term visas (e.g., individuals on student, work, and working holiday visas who migrated to the UK within the last 5 years) in the Million Migrant-NIMS cohort to estimate how international emigration could affect our estimates for overdue vaccinations (see details in Appendix F). R version 4.1.2 was used for analyses.

### Ethics

Surveillance of COVID-19 vaccination is undertaken under Regulation 3 of the Health Service (Control of Patient Information) Regulations 2002 to collect confidential patient information under Sections 3(1) (a)–(c), 3(1)(d) (i) and (ii), and 3(3). This study protocol was subject to regulatory review by UKHSA and was found to be fully compliant with all regulatory requirements. As part of International Organization for Migration health screening processes, non-EU migrants and refugees consented for their data to be used by relevant UK authorities and agencies to guide service improvement. There was no participant compensation.

### Reporting summary

Further information on research design is available in the Nature Portfolio Reporting Summary linked to this article.

### Supplementary information

Supplementary Information Appendix A. Further details on the UK COVID-19 vaccination programme. Appendix B. UK TB pre-entry screening programme and refugee resettlement schemes. UK TB pre-entry screening programme. UK refugee resettlement schemes. Appendix C. Linkage methodology. Step 1: linking the Million Migrant cohort to NHS PDS. Step 2: linking the Million Migrant cohort to NIMS COVID-19 vaccination records. Appendix D. Defining data sources, study outcomes and variables. Description of data sources. Defining study outcomes. Variables. Appendix E. Theoretical diagrams and confounder selection. Explaining confounder selection and models. Appendix F. Sensitivity analyses. Sensitivity analysis 1: estimating impact of emigration and embarkations on overdue second doses. Appendix G. Results—data flow diagram and linkage. Appendix H. Results—delayed vaccination. Appendix I. Results—overdue vaccination. References.

Reporting Summary

## Data Availability

The data that support the findings of this study are held by UKHSA and OHID within the UK government. Access to the data is currently restricted and not publicly available. Researchers interested in working with these datasets should contact these agencies directly. The authors (R.B.) will apply for approval to create an anonymized version of the data by 2024 available upon request for research and replication purposes, provided that the request come from individuals affiliated with a university. If approved, the data will be held in the UCL Data Safe Haven and will be maintained until 2034. OpenSAFELY dataset is publicly available (https://reports.opensafely.org/reports/vaccine-coverage-index/).

## Code availability

Our scripts for data processing and transformation are available for inspection in our public GitHub repository https://github.com/rburns520/COVID-19-vaccination-in-the-Million-Migrant-cohort/releases/tag/v1.0 and https://zenodo.org/doi/10.5281/zenodo.10519055.
