# Author Correction: COVID-19 vaccination coverage for half a million non-EU migrants and refugees in England

**DOI:** 10.1038/s41562-024-01845-4

**Published:** 2024-02-19

**Authors:** Rachel Burns, Sacha Wyke, Max T. Eyre, Yamina Boukari, Tina B. Sørensen, Camille Tsang, Colin N. J. Campbell, Sarah Beale, Dominik Zenner, Sally Hargreaves, Ines Campos-Matos, Katie Harron, Robert W. Aldridge

**Affiliations:** 1 Centre for Public Health Data Science, Institute of Health Informatics, University College London, London, UK; 2 Department of Health and Social Care, Office for Health Improvement and Disparities, London, UK; 3 Department of Disease Control, Faculty of Infectious and Tropical Diseases, London School of Hygiene and Tropical Medicine, London, UK; 4 Immunisation and Vaccine Preventable Diseases, UK Health Security Agency, London, UK; 5 Institute of Epidemiology and Health Care, University College London, London, UK; 6 Global Public Health Unit, Wolfson Institute of Population Health, Barts and The London School of Medicine and Dentistry, Queen Mary University of London, London, UK; 7 Infection and Population Health Department, Institute of Global Health, University College London, London, UK; 8 Institute for Infection and Immunity, St George’s University of London, Cranmer Terrace London, London, UK; 9 UK Health Security Agency, London, UK; 10 UCL Great Ormond Street, Institute of Child Health, University College London, London, UK

**Keywords:** Epidemiology, Health services

Correction to: *Nature Human Behaviour* 10.1038/s41562-023-01768-6, published online 4 December 2024.

In the version of the article initially published, the Code Availability section contained an outdated link and has now been amended to read “Our scripts for data processing and transformation are available for inspection in our public GitHub repository https://github.com/rburns520/COVID-19-vaccination-in-the-Million-Migrant-cohort/releases/tag/v1.0 and 10.5281/zenodo.10519055”. This has been corrected in the HTML and PDF versions of the article.

