# Supplementary material for: COVID-19 vaccination coverage for half a million non-EU migrants and refugees in England
Source: Nat Hum Behav. 2023 Dec 4;8(2):288–99. doi: 10.1038/s41562-023-01768-6 (PMC10896718; doi:10.1038/s41562-023-01768-6)
Supplement: Supplementary file 1 — Appendix A. Further details on the UK COVID-19 vaccination programme. Appendix B. UK TB pre-entry screening programme and refugee resettlement schemes. UK TB pre-entry screening programme. UK refugee resettlement schemes. Appendix C. Linkage methodology. Step 1: linking the Million Migrant cohort to NHS PDS. Step 2: linking the Million Migrant cohort to NIMS COVID-19 vaccination records. Appendix D. Defining data sources, study outcomes and variables. Description of data sources. Defining study outcomes. Variables. Appendix E. Theoretical diagrams and confounder selection. Explaining confounder selection and models. Appendix F. Sensitivity analyses. Sensitivity analysis 1: estimating impact of emigration and embarkations on overdue second doses. Appendix G. Results—data flow diagram and linkage. Appendix H. Results—delayed vaccination. Appendix I. Results—overdue vaccination. References. [file 41562_2023_1768_MOESM1_ESM.pdf]

nature human behaviour

Article https://doi.org/10.1038/s41562-023-01768-6

COVID-19 vaccination coverage for half a million non-EU migrants and refugees in England

In the format provided by the authors and unedited

Supplementary Appendix: COVID-19 vaccination coverage for half a million non-EU migrants and refugees in England

Contents

Appendix A. Further details on the UK COVID-19 vaccination programme

Appendix B. UK tuberculosis pre-entry screening programme and refugee resettlement schemes

- UK Tuberculosis Pre-entry Screening Programme
- UK Refugee Resettlement Schemes

Appendix C. Linkage Methodology

- Step 1: Linking the Million Migrant cohort to NHS PDS
- Step 2. Linking the Million Migrant cohort to NIMS COVID-19 Vaccination records

Appendix D. Defining data sources, study outcomes, and variables

- Description of data sources
- Defining study outcomes
- Variables

Appendix E. Theoretical diagrams and confounder selection

- Explaining confounder selection and models

Appendix F. Sensitivity analyses

- Sensitivity analysis 1: estimating impact of emigration and embarkations on overdue second doses

Appendix G. Results - Data flow diagram and linkage

Appendix H. Results - Delayed vaccination

Appendix I. Results - Overdue vaccination

References

Appendix A. Further details on the UK COVID-19 vaccination programme

The UK COVID-19 vaccination programme started on the 8th of December 2020 and was divided into three phases (Supplementary Table 1). The vaccine was first offered to pre-defined priority groups and then rolled out to the entire adult population.[1]

Supplementary Table 1. UK COVID-19 vaccination programme by phase, timeline, and priority groups.

Phase	Timeline	Priority group
1	Started 08/12/2020	1. Residents in care homes for older adults and cares
		2. Individuals 80 years of age and over
		Front-line health and social care workers
		3. Individuals 75 years of age and older
		4. Individuals 70 years of age and older
		Clinically extremely vulnerable individuals
		5. Individuals 65 years of age and older
		6. Individuals aged 16-64 with underlying health conditions
		Unpaid carers
		7. Individuals 60 years of age and over
		8. Individuals 55 years of age and over
		9. Individuals 50 years of age and over
2	Started 04/2021	10. Individuals between 40-49 years
		11. Individuals between 30-39 years
		12. Individuals between 18-29 years
3	Started 09/2021	Boosters
	08/2021	Individuals between 16-17 years-old
	09/2021	Individuals between 12-15 years old

COVID-19 vaccine mandates

COVID-19 vaccination mandates were in place for health and social care staff in England from November 2021 through 15 March 2022.[2] The UK government amended the Health and Social Care Act 2008 to mandate COVID-19 vaccination for healthcare workers unless exempt. This mandate was revoked by the government on 15 March 2022.

Integrating COVID-19 vaccinations received abroad into UK COVID-19 NIMS dataset

In the first instance, people living in England who received their vaccine from a different devolved administration (e.g. Welsh or Scottish borders) were added into NIMS. Secondly, from September 2021, UK recognised the following vaccines Covidshield, Bharat Biotect, SinoPharm, SinoVac given in other countries and were recorded in NIMS. Our cohort includes only individuals on student visa who arrived prior to 2020.

Appendix B. UK tuberculosis pre-entry screening programme and refugee resettlement schemes

UK Tuberculosis Pre-entry Screening Programme

All individual's resident in a non-European Country where tuberculosis is common (40 cases per 100,000 people) and planning to come and live in the UK for more than 6 months are screened for tuberculosis as part of the UK visa application process.[3] The UK tuberculosis pre-entry screening programme was first piloted in 15 countries from 2005 and then subsequently rolled out to 101 countries through four phases between May 2012 and March 2014. The non-EU migrant pre-entry tuberculosis dataset included individuals screened between 1 January 2005 and 31 December 2020.

Supplementary Table 2. List of countries in the UK tuberculosis pre-entry screening programme and rollout date.

Country	Pre-entry screening rollout phase
Cambodia	Pre-entry pilot scheme
United Republic of Tanzania	Pre-entry pilot scheme
Bangladesh	Pre-entry pilot scheme
Kenya	Pre-entry pilot scheme
Eritrea	Pre-entry pilot scheme
Pakistan	Pre-entry pilot scheme
Sudan	Pre-entry pilot scheme
Ivory Coast	Pre-entry pilot scheme
Niger	Pre-entry pilot scheme
Laos	Pre-entry pilot scheme
Thailand	Pre-entry pilot scheme
Burkina Faso	Pre-entry pilot scheme
Ghana	Pre-entry pilot scheme
Somalia	Pre-entry pilot scheme
Togo	Pre-entry pilot scheme
Malaysia	Phase 1 - completed 31/12/12
India	Phase 1 - completed 31/12/12
South Africa	Phase 1 - completed 31/12/12
Philippines	Phase 1 - completed 31/12/12
Mali	Phase 1 - completed 31/12/12
Lesotho	Phase 1 - completed 31/12/12
Uganda	Phase 2 - completed 31/10/13
Morocco	Phase 2 - completed 31/10/13
Indonesia	Phase 2 - completed 31/10/13
Zambia	Phase 2 - completed 31/10/13
Zimbabwe	Phase 2 - completed 31/10/13
Malawi	Phase 2 - completed 31/10/13
Vietnam	Phase 2 - completed 31/10/13
Sierra Leone	Phase 2 - completed 31/10/13
Gambia	Phase 2 - completed 31/10/13
Ethiopia	Phase 2 - completed 31/10/13
Russia	Phase 3 - completed 31/12/13
Senegal	Phase 3 - completed 31/12/13
Mauritania	Phase 3 - completed 31/12/13
Mongolia	Phase 3 - completed 31/12/13
Mozambique	Phase 3 - completed 31/12/13
East Timor	Phase 3 - completed 31/12/13
Central African Republic	Phase 3 - completed 31/12/13
Tajikistan	Phase 3 - completed 31/12/13
Republic of Congo	Phase 3 - completed 31/12/13
Moldova	Phase 3 - completed 31/12/13
Namibia	Phase 3 - completed 31/12/13
Botswana	Phase 3 - completed 31/12/13

Country	Pre-entry screening rollout phase
Solomon Islands	Phase 3 - completed 31/12/13
Ukraine	Phase 3 - completed 31/12/13
Angola	Phase 3 - completed 31/12/13
Uzbekistan	Phase 3 - completed 31/12/13
South Korea	Phase 3 - completed 31/12/13
Burundi	Phase 3 - completed 31/12/13
Liberia	Phase 3 - completed 31/12/13
Papua New Guinea	Phase 3 - completed 31/12/13
Nepal	Phase 3 - completed 31/12/13
Haiti	Phase 3 - completed 31/12/13
Peru	Phase 3 - completed 31/12/13
Nigeria	Phase 3 - completed 31/12/13
Myanmar	Phase 3 - completed 31/12/13
North Korea	Phase 3 - completed 31/12/13
Afghanistan	Phase 3 - completed 31/12/13
Suriname	Phase 3 - completed 31/12/13
Democratic Republic of the Congo	Phase 3 - completed 31/12/13
Ecuador	Phase 3 - completed 31/12/13
Equatorial Guinea	Phase 3 - completed 31/12/13
Kazakhstan	Phase 3 - completed 31/12/13
Gabon	Phase 3 - completed 31/12/13
Bhutan	Phase 3 - completed 31/12/13
Madagascar	Phase 3 - completed 31/12/13
Guinea Bissau	Phase 3 - completed 31/12/13
Chad	Phase 3 - completed 31/12/13
Cameroon	Phase 3 - completed 31/12/13
China	Phase 3 - completed 31/12/13
Guinea	Phase 3 - completed 31/12/13
Rwanda	Phase 3 - completed 31/12/13
Djibouti	Phase 3 - completed 31/12/13
Guyana	Phase 3 - completed 31/12/13
Kyrgyzstan	Phase 3 - completed 31/12/13
Swaziland	Phase 3 - completed 31/12/13
Bolivia	Phase 3 - completed 31/12/13
Iraq	Phase 4 - completed 31/03/14
Guatemala	Phase 4 - completed 31/03/14
Benin	Phase 4 - completed 31/03/14
Dominican Republic	Phase 4 - completed 31/03/14
Georgia	Phase 4 - completed 31/03/14
Azerbaijan	Phase 4 - completed 31/03/14
Brunei	Phase 4 - completed 31/03/14
Belarus	Phase 4 - completed 31/03/14
South Sudan	Phase 4 - completed 31/03/14
Sri Lanka	Phase 4 - completed 31/03/14
Panama	Phase 4 - completed 31/03/14
Vanuatu	Phase 4 - completed 31/03/14
Paraguay	Phase 4 - completed 31/03/14
Armenia	Phase 4 - completed 31/03/14
Turkmenistan	Phase 4 - completed 31/03/14
Algeria	Phase 4 - completed 31/03/14

UK Refugee Resettlement Schemes

The UK government accepts refugees identified by the United Nations High Commissioner for Refugees (UNHCR) under a series of resettlement schemes. These schemes include: the Gateway Protection Programme, the Mandate Resettlement Scheme, the Syrian Vulnerable Persons Resettlement Scheme, and the Vulnerable Children Resettlement Scheme.[4] These individuals receive a pre-entry health assessment prior to arrival in the UK

that aims to identify health and social care needs to be addressed and accommodated for once in the UK.[5] The refugee pre-arrival dataset used includes individuals who were given a health assessment between 1 March 2013 and 31 December 2022. The cohort does not include individuals on Afghan Citizens Resettlement Scheme (ACRS), Afghan Relocations and Assistance Policy (ARAP) or Ukrainian resettlement schemes.

Appendix C. Linkage Methodology

Step 1: Linking the Million Migrant cohort to NHS PDS

The Million Migrant cohort records were deterministically linked to the NHS PDS (via the PDS tracing service) to obtain NHS numbers and UK postcodes. Only exact matches between all linkage variables were retrieved. Adjustments to the linkage variables were made in order to account for differences in name structure or errors in recorded date of birth (Supplementary Table 3).

Supplementary Table 3. Modifications to linkage variables by round in exact deterministic linkage of Million Migrant cohort to NHS PDS.

Round	Variable	Modification
1	Forename	Accented characters transformed into base unaccented version; hyphens and apostrophes replaced with a wildcard (*)
	Surname	Accented characters transformed into base unaccented version; hyphens and apostrophes replaced with a wildcard (*)
2	Forename	Placed a wildcard (*) after the first forename (e.g. Anna* if Anna Marie).
	Surname	If more than one surname, placed a wildcard (*) in front of the last surname (e.g. *Smith if Roberts Smith).
3	Forename	Placed a wildcard (*) after all forenames (e.g. Anna* if Anna Marie or Anna).
4	Date of birth	Exchanged month and day.

Step 2. Linking the Million Migrant cohort to NIMS COVID-19 Vaccination records

The Million Migrant cohort was linked to NIMS COVID-19 vaccination records using a stepwise deterministic matching procedure developed by NHS Digital (Supplementary Table 4). Match rank corresponds to the step at which the match will be determined and demonstrates the quality of the matching. For example, a lower match rank value indicates a match based on a greater number of restrictions and is considered to be stronger evidence of a true positive match.

Supplementary Table 4. Stepwise deterministic matching algorithm according to detail of identifying variables for linkage of Million Migrant cohort to NIMS data.

Match rank	NHS Number	Date of birth	Sex	Postcode
1	Exact	Exact	Exact	Exact
2	Exact	Exact	Exact	
3	Exact	Partial	Exact	Exact
4	Exact	Partial	Exact	
5	Exact			Exact
6*		Exact	Exact	Exact
7**		Exact	Exact	Exact
8***		Exact	Exact	Exact

* Where NHS number does not contradict the match and DOB is not 1 January and the postcode is in the 'ignore' list such as postcodes for communal establishments such as hospitals, care homes, prisons, and boarding schools.
** Where NHS number does not contradict the match and DOB is not 1 January.
*** Where DOB is not 1 January.

Appendix D. Defining data sources, study outcomes, and variables

Description of data sources

Supplementary Table 5. Data sources and variables used in the study

Dataset	Collected by	Dataset details	Variables
**Non-EU migrant pre-entry tuberculosis screening dataset**	IOM, held by UKHSA	Derived from records on non-EU migrants' pre-entry tuberculosis screening collected between 1 January 2005 and 31 December 2020. Part of the Million Migrant cohort.	Age, sex, region of origin, visa type, and year of screening
**Refugee pre-arrival health assessment dataset**	IOM, held by UKHSA	Derived from records on refugees' pre-arrival health assessment collected between 1 January 2013 and 31 December 2020. Part of the Million Migrant cohort.	Age, sex, region of origin, visa type, and year of health assessment
**NHS Personal Demographic Service**	NHS	National database of patient demographic information in England used to identify the NHS numbers and postcode in the Million Migrant cohort.	NHS number, UK postcode
**NIMS COVID-19 vaccination dataset**	UKHSA	Records on NHS COVID-19 vaccination programme in England.	COVID-19 vaccine dose, COVID-19 vaccine dose date, ethnicity
**OpenSAFELY**	The OpenSAFELY Collaborative	Participants included all patients (N = 19,188,764; migrants and non-migrants) with a recorded date of birth and sex from 40% of general practices that use TPP SystmOne software in England. Publicly available data with COVID-19 vaccine dose aggregated for each day by age group and ethnicity.	COVID-19 vaccine dose (aggregated by day), ethnicity (available only >=18), age group

Defining study outcomes

Delayed vaccination

Vaccination delay was a binary variable used to investigate differences in the timing of uptake of second and third vaccination by age and visa type. Individuals aged 18 and older who had completed at least 14 weeks of follow up after their last recorded COVID-19 vaccine dose by the end of the study period (20 April 2022) were included in this outcome. Second and third doses given less than 19 days after the first or second dose, were excluded from the denominator population to follow the same perimeters as the OpenSAFELY cohort (270 second doses and 112 third doses excluded within the Million Migrant-NIMS cohort).[6]

Second doses were considered delayed if they were not received within 14 weeks after the first dose. This was in line with the recommendation for most adults to receive their second dose within 12 weeks of their first dose with an additional two weeks to account for appointment bookings.[7] Please note that the intervals between the first and second doses changed over time in England. Although a minority of participants in our cohort were eligible for their second dose before the 12-week interval, we decided to use the 12-week interval as the cut off for a delayed vaccine after which all participants were always able to receive a second dose.

Based on the UK booster vaccine rollout schedule, a booster programme was rolled out in September 2022 which for most of the population will be a third dose. Please note that when referring to a third dose in this study, this relates to the initial booster programme and not a third primary dose for those who are severely

immunocompromised. A third dose was considered delayed if it was not received within 30 days following each age group's eligible date. Dates were: 29 September 2021 for adults over 50 years at least six months post second dose, 7 December 2021 for adults over 40 years at least three months post second dose, and 28 December 2021 for adults under 40 years with at least three months post second dose.[8–10] If an individual did not have the minimum required months post second dose at the time of their age group's eligible date, their third dose was assessed within 30 days after they fulfilled the required time period. To account for change in the booster eligibility criteria from 6 to 3 months on 13 December 2021, adults over the age of 18 who received a second dose from 13 September 2021 were considered eligible to receive a booster from 3 months post second dose.[11]

Variables

Million Migrant cohort

Sex, region of origin, arrival visa type, and year of arrival to England were available from the pre-entry TB screening and refugee pre-arrival health assessment datasets. Region of origin was determined by country of origin and categorised into the following World Bank geographical areas: East Asia and Pacific, Europe and Central Asia, Latin America and Caribbean, Middle East and North Africa, North America, South Asia, Sub-Saharan Africa and Missing. Arrival visa type was composed of the following categories: Students, Work, Settlement and Dependents, Family Reunion, Refugee, Other, and Missing. The Other category includes a range of individuals on UK visa agreements to specific migration situations. Year of arrival to England was assumed to be equivalent to the year of migrant pre-entry TB screening or refugee pre-arrival health assessment record. Region resident in England was derived from the Million Migrant cohort following linkage to PDS for attainment of NHS numbers. Region of residence in England was determined from the UK postcode. Ethnicity was derived from the Million Migrant-PDS cohort following linkage to the COVID-19 NIMS dataset. Ethnicity categories were based on the 2011 Census' 18 categories grouped into 6 broader categories. These 6 broader categories include Asian, Black, Mixed, White, Other and Unknown. The Other category included individuals with Arab, any other ethnic group, Chinese (other), and any other Asian background. The Unknown category was anyone with a missing ethnicity category. Age groups were calculated from the individuals' age at first dose and were the following categories: 16-17, 18-29, 30-39, 40-49, 50-54, 55-59, 60-64, 65-69, 70-79, 80+.

England cohort

The OpenSAFELY cohort was publicly available aggregated data. Daily vaccination count data was aggregated by age group and ethnicity. Age groups were the same categories as the Million Migrant cohort. Ethnicity was extracted from primary care records and used the same categories as the Million Migrant cohort listed above.

Appendix E. Theoretical diagrams and confounder selection

A

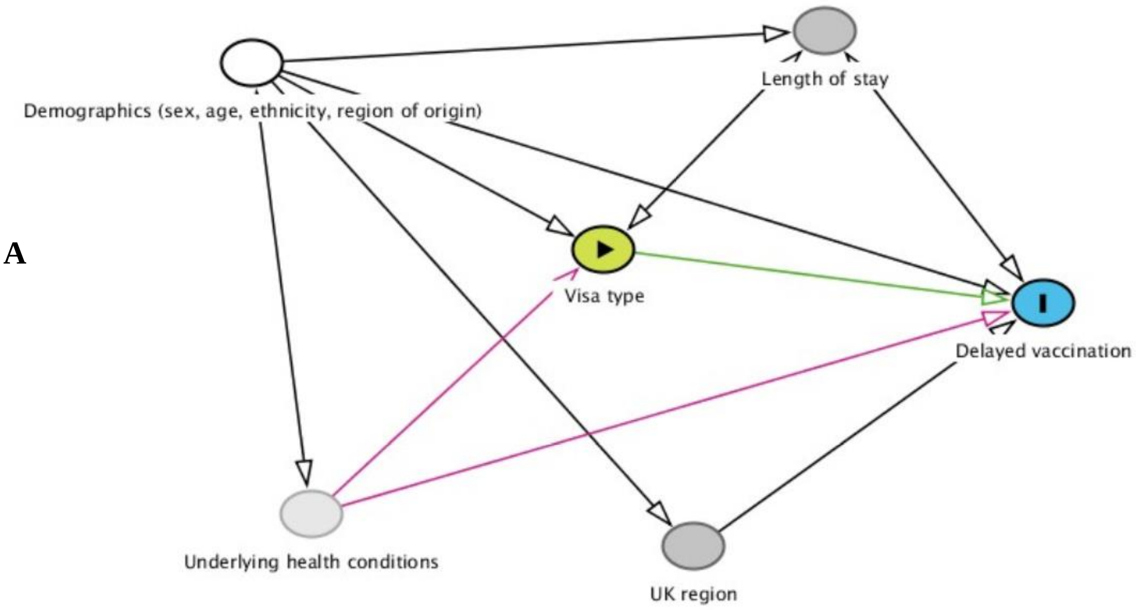

B

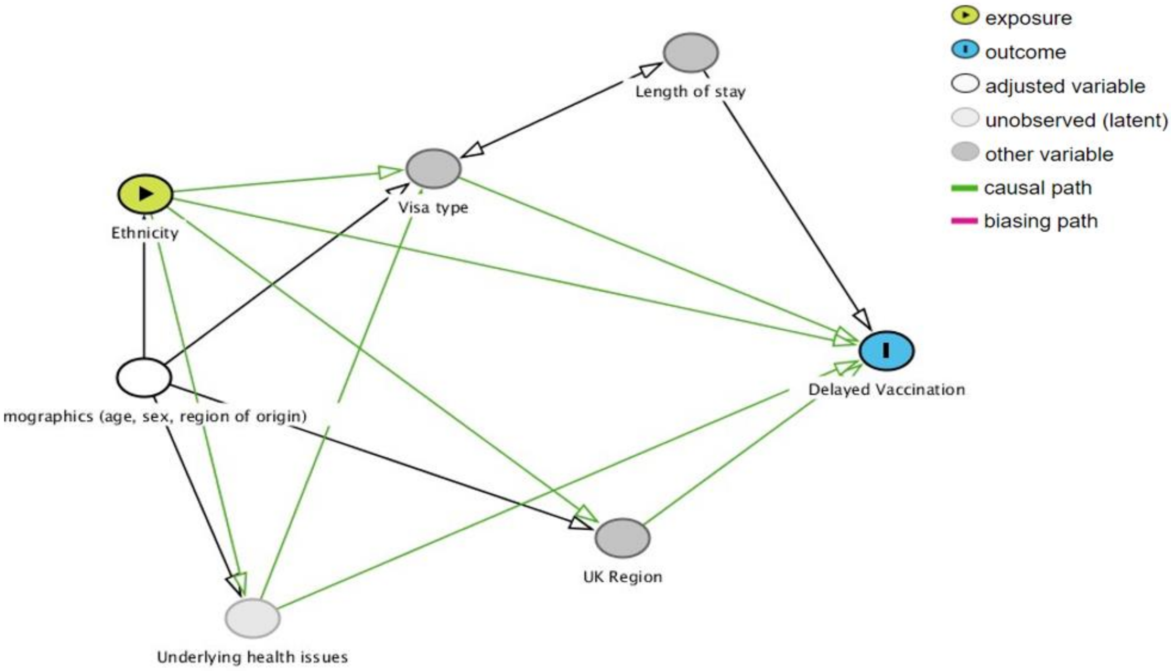

Supplementary Figure 1. Directed acyclic graph of the relationship between visa type and delayed vaccination (A) and ethnicity and delayed vaccination (B).

Explaining confounder selection and models

The multivariable logistic regression models were performed to describe the association between each delayed vaccination dose and exposure (either visa type or ethnicity). Although these analyses were not performed to describe a causal relationship between the exposure and outcome, we drew a directed acyclic graph (DAG) of variables that are related to both to help guide the selection of confounders (Supplementary Figure 1).

We conducted model checks for multicollinearity between region of origin, ethnicity, and visa type by calculating the generalised variance inflation factor (GVIF) using the vif commend in the car package. Evidence of multicollinearity was assumed if the adjusted GVIF exceeded 10.

With visa type as the main exposure, we adjusted the final model by age, sex, and ethnicity. Region of origin was not included as it showed high multicollinearity with ethnicity. With ethnicity as the main exposure, we adjusted the final model by age and sex, as visa type is likely to be on the causal pathway between the exposure and outcome.

For second dose delayed, only individuals aged 18 years and older who had received their second dose within the study period were included. For third dose delayed, only individuals aged 18 years and older who had received their third dose within the study period were included.

Appendix F. Sensitivity analyses

Sensitivity analysis 1: estimating impact of emigration and embarkations on overdue second doses

As our dataset does not contain information on emigration or embarkations out of England, our estimates for overdue vaccinations could have been biased by overestimating the denominator and therefore the total proportion of people not receiving their second or third dose during the study follow up period. Given that some populations are likely to be more mobile, the impact of this bias is unlikely to be uniform across migrant sub-groups. It is important that we carried out some sensitivity analyses assuming different scenarios to estimate how this bias may affect the results of the study.

First, we restricted the study follow up period to end on 1 June 2021 instead of 20 April 2022, with an aim to estimate the impact of movement out of England once international travel had resumed on the proportion of overdue second vaccinations. Individuals who had received their last recorded dose at least 14 weeks before 1 June 2021 were included in this sensitivity analysis.

Second, we removed all individuals on shorter term visas (student, work, working holiday) who migrated to the UK within the last 5 years (after 2017) from the Million Migrant-NIMS cohort (45143/467904, 9.6%). This group is the most likely to have emigrated during the study period given their shorter term visas and total length of time they had been living in England. This was done to examine a scenario in which the proportion of the cohort mostly likely to emigrate had returned to their region of origin during the study follow up period. We examined the impact of the remaining proportion of the cohort on the proportions of overdue second vaccinations.

Appendix G. Results - Data flow diagram and linkage

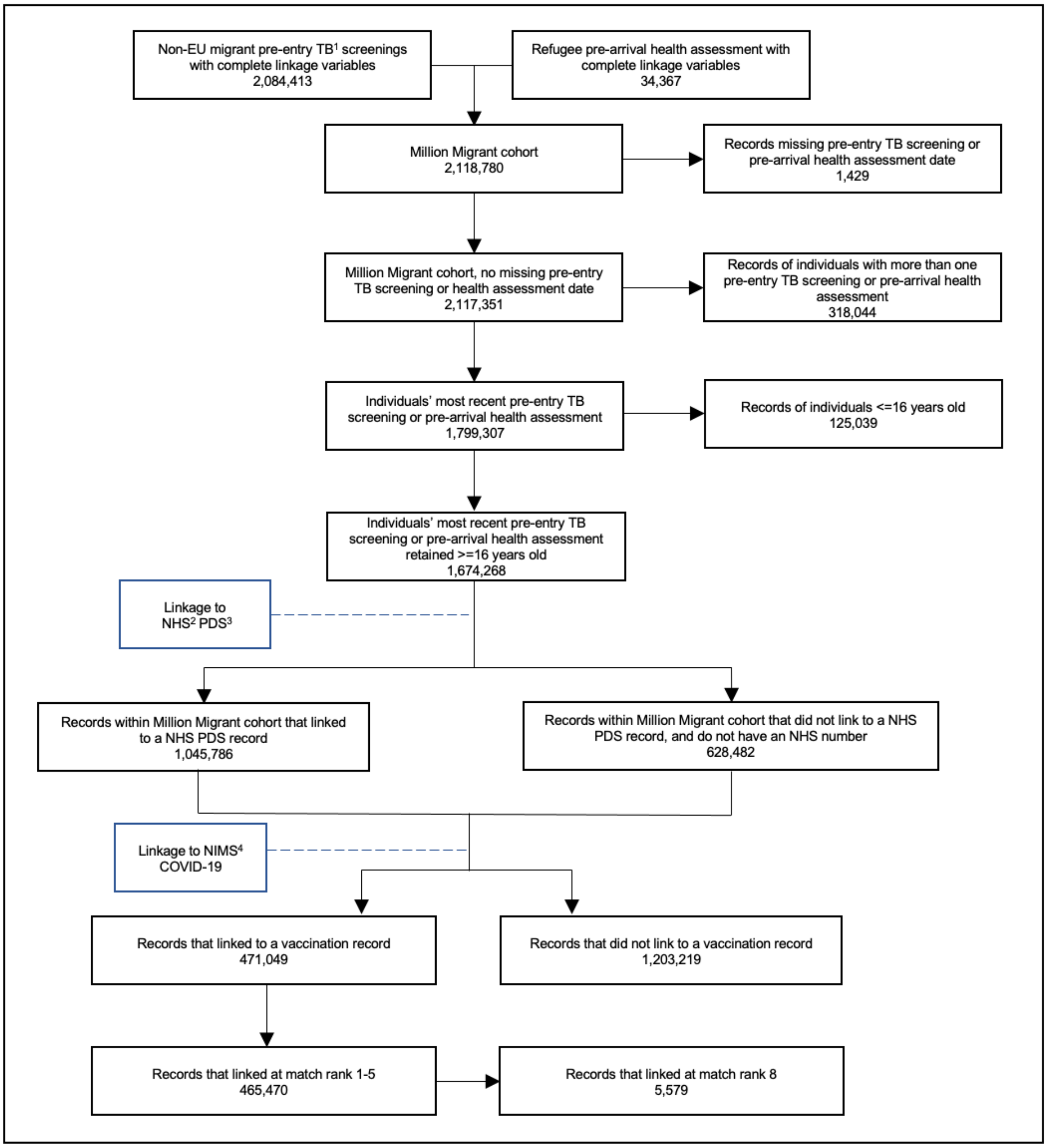

Supplementary Figure 2. Data flow diagram; TB = Tuberculous; NHS = National Health Service; PDS = Personal Demographic Service; NIMS = National Immunisation Management Service

Supplementary Table 6. Characteristics of individuals in the Million Migrant cohort >=16 years old who matched or did not match in NIMS by match rank.

Characteristic	Did not match N = 1,203,219 (73.1%)	Matched N = 471,049 (26.9%)	Match Rank 1 N = 342,140 (72.6%)	2 N = 114,568 (24.3%)	3 N = 3,577 (0.8%)	4 N = 2,405 (0.5%)	5 N =2,780 (0.6%)	8 N = 5,579 (1.2%)
**Sex**								
Female	562,714 (68.1%)	263,741 (31.9%)	192,755 (73.1%)	63,064 (23.9%)	2,045 (0.8%)	1,458 (0.6%)	1,202 (0.5%)	3,217 (1.2%)
Male	640,505 (75.5%)	207,308 (24.5%)	149,385 (72.1%)	51,504 (24.8%)	1,532 (0.7%)	947 (0.5%)	1,578 (0.8%)	2,362 (1.1%)
**Age**	31 (26, 38)	32 (26, 38)	33 (28, 39)	29 (24, 35)	29 (23, 35)	24 (21, 30)	28 (22, 35)	24 (22, 30)
**Age group**								
16-17	13,396 (66.1%)	6,866 (33.9%)	5,118 (74.5%)	1,469 (21.4%)	73 (1.1%)	26 (0.4%)	52 (0.8%)	128 (1.9%)
18-29	498,237 (74.5%)	170,601 (25.5%)	105,631 (61.9%)	55,924 (32.8%)	1,757 (1.0%)	1,772 (1.0%)	1,526 (0.9%)	3,991 (2.3%)
30-39	450,222 (69.1%)	201,092 (30.9%)	154,572 (76.9%)	42,938 (21.4%)	1,263 (0.6%)	491 (0.2%)	830 (0.4%)	998 (0.5%)
40-49	162,328 (71.1%)	66,105 (28.9%)	54,599 (82.6%)	10,421 (15.8%)	374 (0.6%)	93 (0.1%)	272 (0.4%)	346 (0.5%)
50-54	32,640 (74.9%)	10,959 (25.1%)	9,213 (84.1%)	1,582 (14.4%)	55 (0.5%)	#	#	57 (0.5%)
55-59	21,913 (79.4%)	5,689 (20.6%)	4,815 (84.6%)	788 (13.9%)	30 (0.5%)	11 (0.2%)	22 (0.4%)	23 (0.4%)
60-64	10,530 (75.7%)	3,380 (24.3%)	2,831 (83.8%)	508 (15.0%)	10 (0.3%)	#	#	14 (0.4%)
65-69	4,941 (68.1%)	2,315 (31.9%)	1,942 (83.9%)	353 (15.2%)	#	#	#	11 (0.5%)
70-79	6,375 (66.4%)	3,230 (33.6%)	2,725 (84.4%)	471 (14.6%)	#	#	16 (0.5%)	#
80+	2,637 (76.5%)	812 (23.5%)	694 (85.5%)	114 (14.0%)	#	#	#	#
**Region of origin***								
East Asia & Pacific	279,917 (74.3%)	96,908 (25.7%)	57,135 (59.0%)	31,148 (32.1%)	2,313 (2.4%)	1,924 (2.0%)	1,063 (1.1%)	3,325 (3.4%)
Europe & Central Asia	19,897 (60.9%)	12,775 (39.1%)	8,533 (66.8%)	3,929 (30.8%)	#	#	83 (0.6%)	216 (1.7%)
Latin America & Caribbean	338 (59.4%)	231 (40.6%)	139 (60.2%)	86 (37.2%)	#	#	#	#
Middle East & North Africa	7,662 (40.3%)	11,367 (59.7%)	9,242 (81.3%)	2,000 (17.6%)	12 (0.1%)	#	#	#
North America	95 (37.3%)	160 (62.7%)	96 (60.0%)	61 (38.1%)	#	#	#	#
South Asia	182,051 (42.3%)	248,507 (57.7%)	193,921 (78.0%)	50,591 (20.4%)	1,213 (0.5%)	454 (0.2%)	962 (0.4%)	1,366 (0.5%)
Sub-Saharan Africa	84,773 (45.6%)	101,089 (54.4%)	73,065 (72.3%)	26,750 (26.5%)	27 (0.0%)	19 (0.0%)	627 (0.6%)	601 (0.6%)
**Entry Visa Type**								
Students	696,546 (85.8%)	115,640 (14.2%)	21,281 (77.0%)	5,800 (21.0%)	208 (0.8%)	53 (0.2%)	126 (0.5%)	157 (0.6%)
Work	123,218 (64.6%)	67,378 (35.4%)	25,661 (59.4%)	15,565 (36.0%)	155 (0.4%)	93 (0.2%)	407 (0.9%)	1,342 (3.1%)

Characteristic	Did not match<br>N = 1,203,219<br>(73.1%)	Matched<br>N = 471,049<br>(26.9%)	Match Rank<br>1<br>N = 342,140<br>(72.6%)	2<br>N = 114,568<br>(24.3%)	3<br>N = 3,577<br>(0.8%)	4<br>N = 2,405<br>(0.5%)	5<br>N =2,780<br>(0.6%)	8<br>N = 5,579<br>(1.2%)
Settlement & Dependents	191,414 (48.2%)	205,755 (51.8%)	7,214 (86.5%)	1,056 (12.7%)	#	#	33 (0.4%)	28 (0.3%)
Family Reunion	48,761 (63.8%)	27,625 (36.2%)	169,722 (82.5%)	33,675 (16.4%)	724 (0.4%)	222 (0.1%)	594 (0.3%)	818 (0.4%)
Refugee	12,500 (60.0%)	8,338 (40.0%)	70,899 (61.3%)	36,911 (31.9%)	1,768 (1.5%)	1,718 (1.5%)	1,330 (1.2%)	3,014 (2.6%)
Other	118,557 (73.3%)	43,223 (26.7%)	2,146 (69.4%)	864 (28.0%)	#	#	23 (0.7%)	40 (1.3%)
Unknown	12,223 (79.8%)	3,090 (20.2%)	45,217 (67.1%)	20,697 (30.7%)	705 (1.0%)	312 (0.5%)	267 (0.4%)	180 (0.3%)
**Length of time in UK (yrs)**	3.7 (2.3, 6.1)	3.5 (1.5, 7.6)	21,281 (77.0%)	5,800 (21.0%)	208 (0.8%)	53 (0.2%)	126 (0.5%)	157 (0.6%)
**Length of time**								
<2	204,935 (58.1%)	147,726 (41.9%)	93,283 (63.1%)	51,432 (34.8%)	187 (0.1%)	137 (0.1%)	893 (0.6%)	1,794 (1.2%)
2 to 3	253,445 (70.3%)	107,031 (29.7%)	72,167 (67.4%)	28,206 (26.4%)	2,432 (2.3%)	1,797 (1.7%)	676 (0.6%)	1,753 (1.6%)
4 to 5	213,708 (76.0%)	67,315 (24.0%)	50,211 (74.6%)	14,546 (21.6%)	758 (1.1%)	420 (0.6%)	432 (0.6%)	948 (1.4%)
6 to 7	170,039 (82.6%)	35,918 (17.4%)	29,241 (81.4%)	5,993 (16.7%)	38 (0.1%)	19 (0.1%)	219 (0.6%)	408 (1.1%)
>8	361,092 (76.2%)	113,059 (23.8%)	97,238 (86.0%)	14,391 (12.7%)	162 (0.1%)	32 (0.0%)	560 (0.5%)	676 (0.6%)
**Region in England**								
East Midlands	30,900 (54.7%)	25,575 (45.3%)	18,611 (72.8%)	6,212 (24.3%)	181 (0.7%)	143 (0.6%)	181 (0.7%)	247 (1.0%)
East of England	30,413 (42.5%)	41,120 (57.5%)	30,484 (74.1%)	9,414 (22.9%)	311 (0.8%)	152 (0.4%)	203 (0.5%)	556 (1.4%)
London	127,429 (46.8%)	144,909 (53.2%)	108,269 (74.7%)	32,554 (22.5%)	1,304 (0.9%)	642 (0.4%)	760 (0.5%)	1,380 (1.0%)
North East	18,872 (56.9%)	14,319 (43.1%)	10,444 (72.9%)	3,433 (24.0%)	116 (0.8%)	78 (0.5%)	75 (0.5%)	173 (1.2%)
North West	45,531 (47.9%)	49,435 (52.1%)	38,637 (78.2%)	9,287 (18.8%)	351 (0.7%)	236 (0.5%)	284 (0.6%)	640 (1.3%)
South East	56,699 (45.0%)	69,398 (55.0%)	52,011 (74.9%)	15,672 (22.6%)	578 (0.8%)	249 (0.4%)	318 (0.5%)	570 (0.8%)
South West	26,230 (54.0%)	22,388 (46.0%)	15,776 (70.5%)	5,861 (26.2%)	200 (0.9%)	117 (0.5%)	164 (0.7%)	270 (1.2%)
West Midlands	45,572 (48.1%)	49,188 (51.9%)	37,933 (77.1%)	9,350 (19.0%)	277 (0.6%)	189 (0.4%)	273 (0.6%)	1,166 (2.4%)
Yorkshire and the Humber	45,909 (55.1%)	37,394 (44.9%)	28,991 (77.5%)	7,277 (19.5%)	235 (0.6%)	163 (0.4%)	198 (0.5%)	530 (1.4%)
Other*	23,519 (94.8 %)	1,280 (5.2%)	80 (6.3%)	868 (67.8%)	-	21 (1.6%)	310 (24.2)	-
Missing	123,663 (88.5%)	16,043 (11.5%)	904 (5.6%)	14,640 (91.3%)	24 (0.1%)	415 (2.6%)	14 (0.1%)	46 (0.3%)

Suppressed due to low cell count; Region of origin = Unknown not shown due to low cell count; **Region of England: Other = Crown Dependencies, Scotland, Wales, and Northern Ireland

Supplementary Table 7. Demographic characteristics of the total Million Migrant cohort, Million Migrant cohort without an NHS number, Million Migrant cohort with an NHS number but no NIMS link, and the Million Migrant-NIMS cohort as of 20 April 2022.

	**Million Migrant cohort**<br><br>N = 1,674,268 (100·0%)	**Million Migrant cohort without an NHS number**<br><br>N =628,482 (37·5%)	**Million Migrant cohort with an NHS number**<br><br>N = 1,045,786 (62.5%)	**Million Migrant cohort with NHS number but no NIMS link**<br><br>N = 574,737 (55.0 %)	**Million Migrant-NIMS cohort[1]**<br><br>N = 465,470 (27·8%)
**Sex**					
Female	826,455 (49.4%)	268,555 (42.7%)	557,900 (53.3%)	294,159 (51.2%)	260,524 (56.0%)
Male	847,813 (50.6%)	359,928 (57.3%)	487,886 (46.7%)	280,578 (48.8%)	204,946 (44.0%)
**Age***					
16-17	20,262 (1.2%)	6,070 (1.0%)	14,192 (1.4%)	7,326 (1.3%)	3,688 (0.8%)
18-29	668,838 (39.9%)	184,187 (29.3%)	484,651 (46.3%)	314,050 (54.6%)	159,899 (34.4%)
30-39	651,314 (38.9%)	267,831 (42.6%)	383,483 (36.7%)	182,391 (31.7%)	204,899 (44.0%)
40-49	228,433 (13.6%)	109,211 (17.4%)	119,222 (11.4%)	53,117 (9.2%)	69,767 (15.0%)
50-54	43,599 (2.6%)	25,060 (4.0%)	18,539 (1.8%)	7,580 (1.3%)	11,410 (2.5%)
55-59	27,602 (1.6%)	17,935 (2.9%)	9,667 (0.9%)	3,978 (0.7%)	5,881 (1.3%)
60-64	13,910 (0.8%)	8,332 (1.3%)	5,578 (0.5%)	2,198 (0.4%)	3,452 (0.7%)
65-69	7,256 (0.4%)	3,624 (0.6%)	3,632 (0.3%)	1,317 (0.2%)	2,358 (0.5%)
70-79	9,605 (0.6%)	4,554 (0.7%)	5,051 (0.5%)	1,821 (0.3%)	3,271 (0.7%)
80+	3,449 (0.2%)	1,678 (0.3%)	1,771 (0.2%)	959 (0.2%)	845 (0.2%)
**Region of origin**					
East Asia & Pacific	565,949 (33.8%)	189,124 (30.1%)	376,825 (36.0%)	279,917 (48.7%)	93,583 (20.1%)
Europe & Central Asia	43,955 (2.6%)	11,283 (1.8%)	32,672 (3.1%)	19,897 (3.5%)	12,559 (2.7%)
Latin America & Caribbean	889 (0.1%)	320 (0.1%)	569 (0.1%)	338 (0.1%)	227 (0.0%)
Middle East & North Africa	32,000 (1.9%)	12,971 (2.1%)	19,029 (1.8%)	7,662 (1.3%)	11,301 (2.4%)
North America	307 (0.0%)	52 (0.0%)	255 (0.0%)	95 (0.0%)	159 (0.0%)
South Asia	741,298 (44.3%)	310,740 (49.4%)	430,558 (41.2%)	182,051 (31.7%)	247,141 (53.1%)
Sub-Saharan Africa	289,832 (17.3%)	103,970 (16.5%)	185,862 (17.8%)	84,773 (14.7%)	100,488 (21.6%)
Missing	38 (0.0%)	22 (0.0%)	16 (0.0%)	#	12 (0.0%)
**Entry Visa Type**					
Students	812,186 (48.5%)	355,016 (56.5%)	457,170 (43.7%)	341,530 (59.4%)	112,626 (24.2%)
Work	190,596 (11.4%)	64,788 (10.3%)	125,808 (12.0%)	58,430 (10.2%)	67,198 (14.4%)
Settlement & Dependents	397,169 (23.7%)	120,831 (19.2%)	276,338 (26.4%)	70,583 (12.3%)	204,937 (44.0%)
Family Reunion	76,386 (4.6%)	25,213 (4.0%)	51,173 (4.9%)	23,548 (4.1%)	27,468 (5.9%)
Refugee	20,838 (1.2%)	8,687 (1.4%)	12,151 (1.2%)	3,813 (0.7%)	8,310 (1.8%)

	**Million Migrant cohort**<br><br>N = 1,674,268 (100·0%)	**Million Migrant cohort without an NHS number**<br><br>N =628,482 (37·5%)	**Million Migrant cohort with an NHS number**<br><br>N = 1,045,786 (62.5%)	**Million Migrant cohort with NHS number but no NIMS link**<br><br>N = 574,737 (55.0 %)	**Million Migrant-NIMS cohort[1]**<br><br>N = 465,470 (27·8%)
Other	161,780 (9.7%)	44,684 (7.1%)	117,096 (11.2%)	73,873 (12.9%)	41,881 (9.0%)
Missing	15,313 (0.9%)	9,263 (1.5%)	6,050 (0.6%)	2,960 (0.5%)	3,050 (0.7%)
**Length of time in UK* (years)**					
<2	352,661 (21.1%)	81,865 (13.0%)	270,796 (25.9%)	123,070 (21.4%)	101,158 (21.7%)
2 to 3	360,476 (21.5%)	82,267 (13.1%)	278,209 (26.6%)	171,178 (29.8%)	129,314 (27.8%)
4 to 5	281,023 (16.8%)	79,844 (12.7%)	201,179 (19.2%)	133,864 (23.3%)	73,375 (15.8%)
6 to 7	205,957 (12.3%)	129,858 (20.7%)	76,099 (7.3%)	40,181 (7.0%)	43,040 (9.2%)
>8	474,151 (28.3%)	254,648 (40.5%)	219,503 (21.0%)	106,444 (18.5%)	118,583 (25.5%)
**Region of England****					
East Midlands	56,475 (3.4%)	-	56,475 (5.4%)	30,900 (5.4%)	25,328 (5.4%)
East of England	71,533 (4.3%)	-	71,533 (6.8%)	30,413 (5.3%)	40,564 (8.7%)
London	272,338 (16.3%)	-	272,338 (26.0%)	127,429 (22.2%)	143,529 (30.8%)
North East	33,191 (2.0%)	-	33,191 (3.2%)	18,872 (3.3%)	14,146 (3.0%)
North West	94,966 (5.7%)	-	94,966 (9.1%)	45,531 (7.9%)	48,795 (10.5%)
South East	126,097 (7.5%)	-	126,097 (12.1%)	56,699 (9.9%)	68,828 (14.8%)
South West	48,618 (2.9%)	-	48,618 (4.6%)	26,230 (4.6%)	22,118 (4.8%)
West Midlands	94,760 (5.7%)	-	94,760 (9.1%)	45,572 (7.9%)	48,022 (10.3%)
Yorkshire & the Humber	83,303 (5.0%)	-	83,303 (8.0%)	45,909 (8.0%)	36,864 (7.9%)
Other	24,799 (1.5%)	-	24,799 (2.4%)	23,519 (4.0%)	1279 (0.3%)
Missing	768,188 (45.9%)	-	139,706 (13.4%)	123,663 (21.5%)	15,997 (3.4%)
**Ethnicity*****					
Black	-	-	-	-	56,664 (12.2%)
Mixed	-	-	-	-	14,338 (3.1%)
Other	-	-	-	-	114,950 (24.7%)
South Asian	-	-	-	-	179,460 (38.6%)
Unknown	-	-	-	-	74,597 (16.0%)
White	-	-	-	-	25,461 (5.5%)
**COVID-19 dose**					
First	-	-	-	-	465,470 (100.0%)
Second	-	-	-	-	427,073 (91.8%)
Third/Booster	-	-	-	-	238,721 (51.3%)

Suppressed due to low cell count; [1] Million Migrant-NIMS cohort includes those with match rang 1-5. Excluded matched rank 8 (N = 5,579)

Appendix H. Results - Delayed vaccination

Supplementary Table 8. Sample size for delayed second and third COVID-19 dose vaccinations by ethnicity and visa type (as seen in Figure 1 in the main text).

	**Second dose delayed Sample Size (N)**	**Third dose delayed Sample Size (N)**
**Ethnicity**		
Black	50670	22634
Mixed	13149	7818
Other	102953	65895
South Asian	167931	85575
Unknown	63030	32011
White	23652	15509
**Visa type***		
Family	24604	13369
Other	34924	17688
Refugee	7217	3176
Settlement	190774	100288
Students	99137	48053
Work	64729	46868

* Unknown visa type removed due to low numbers.

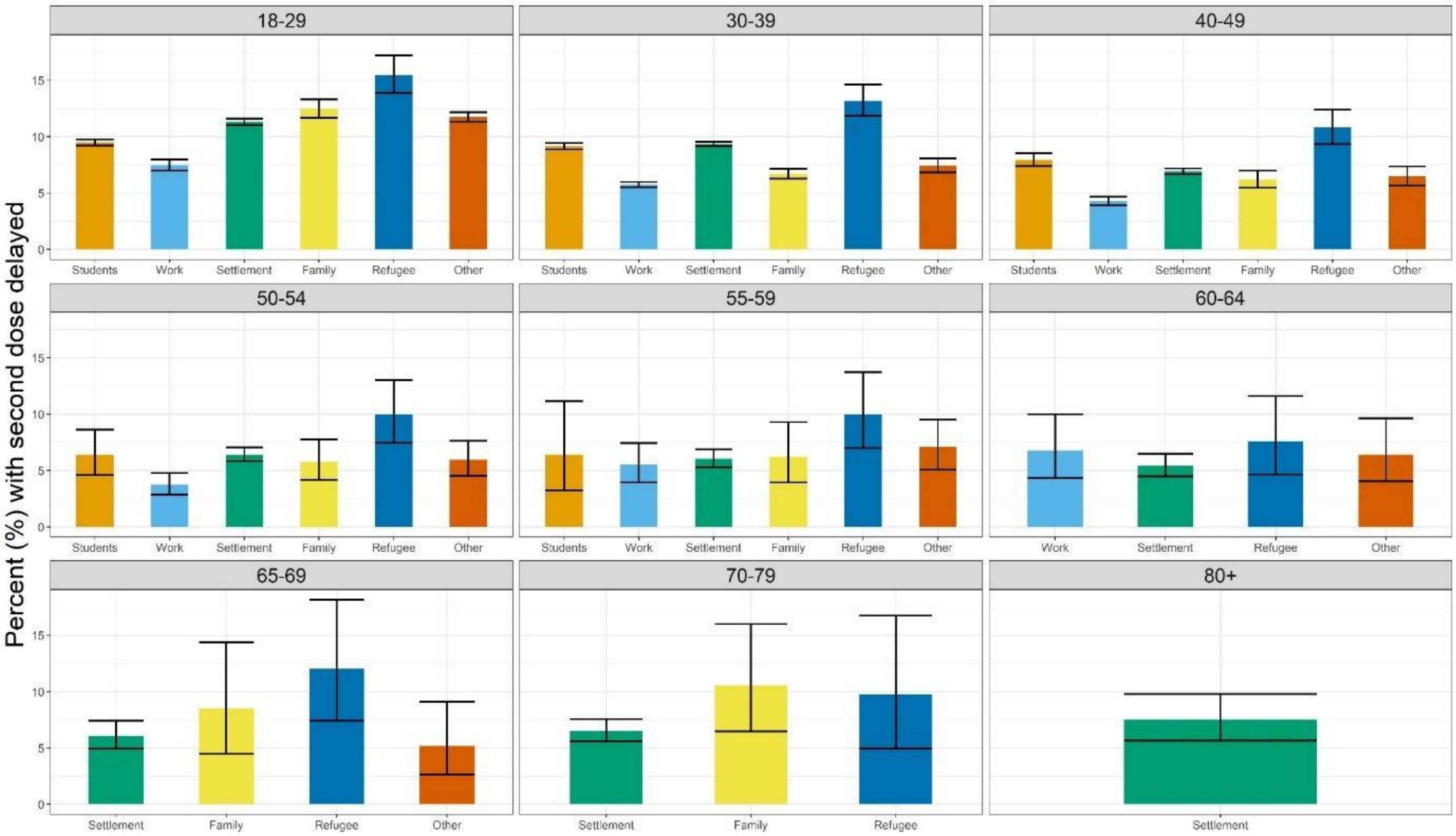

Supplementary Figure 3. **Second dose delayed by visa type and age.**
Proportion (%) and error bars represent 95% Confidence Intervals of second dose delayed by age group comparing visa type as of 20 April 2022.

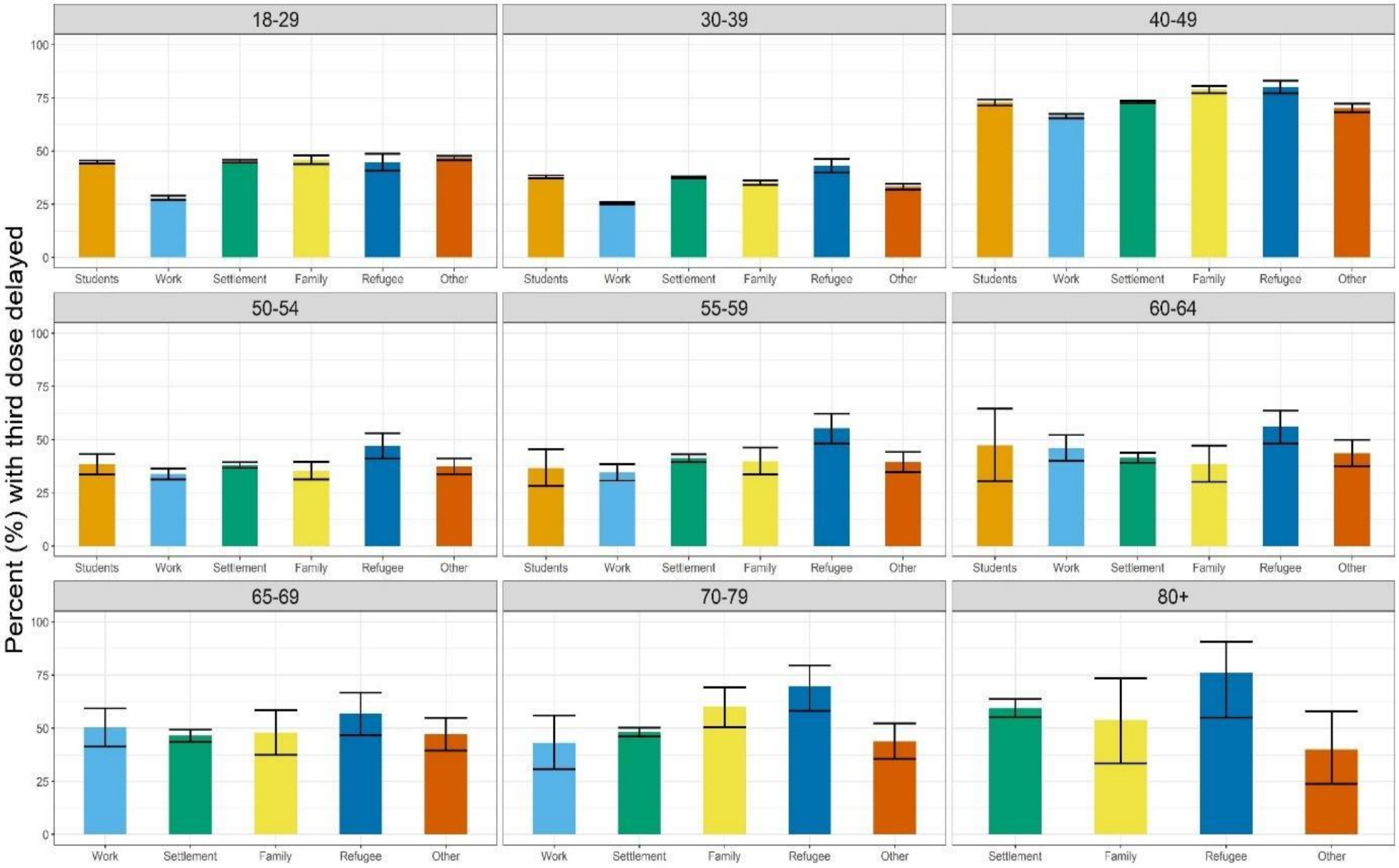

Supplementary Figure 4. **Third dose delayed by visa type and age.**
Proportion (%) and error bars represent 95% Confidence Intervals of third dose delayed by age group comparing visa type as of 20 April 2022.

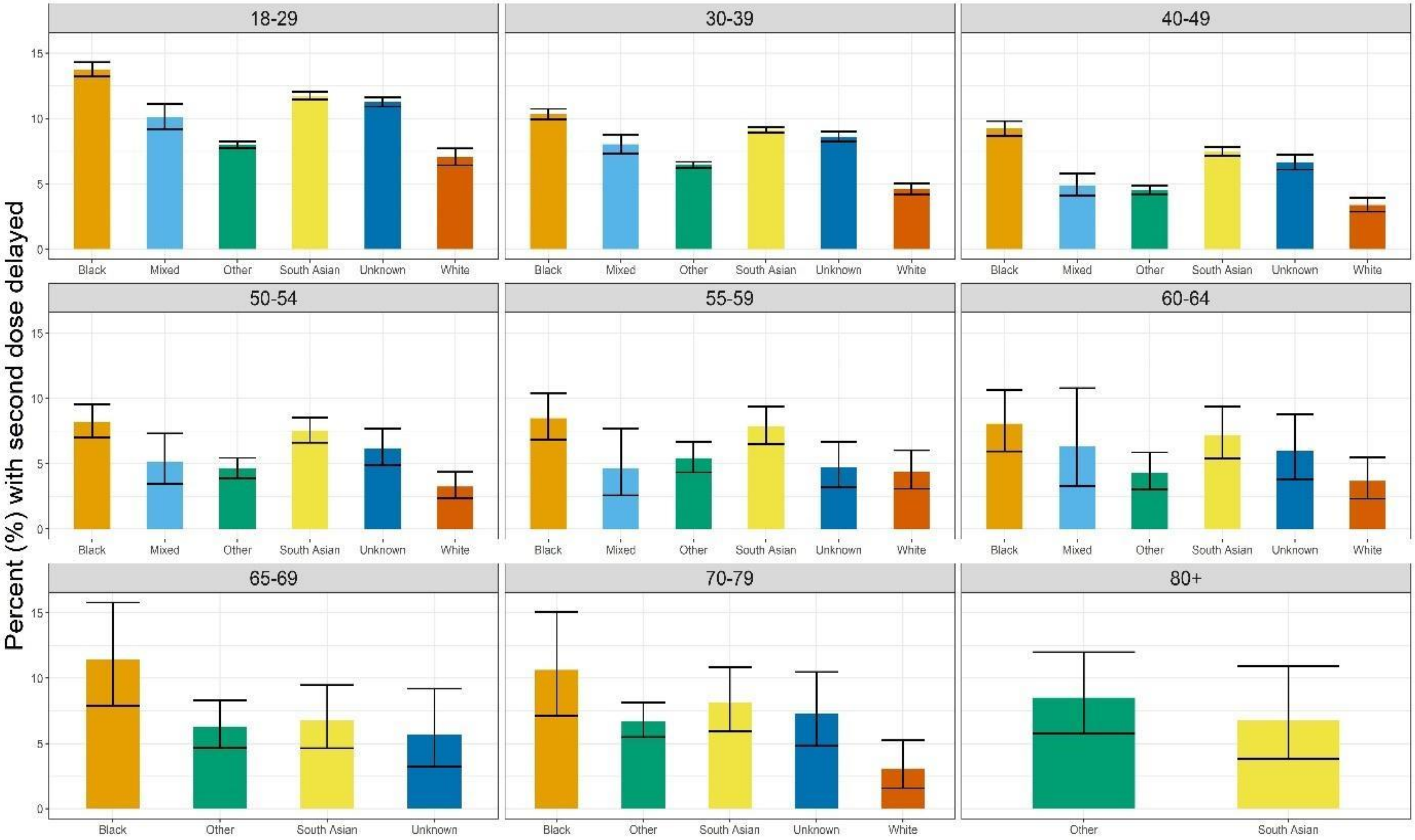

Supplementary Figure 5. Second dose delayed by ethnicity and age.
Proportion (%) and error bars represent 95% Confidence Intervals of second dose delayed by age group comparing ethnicity as of 20 April 2022.

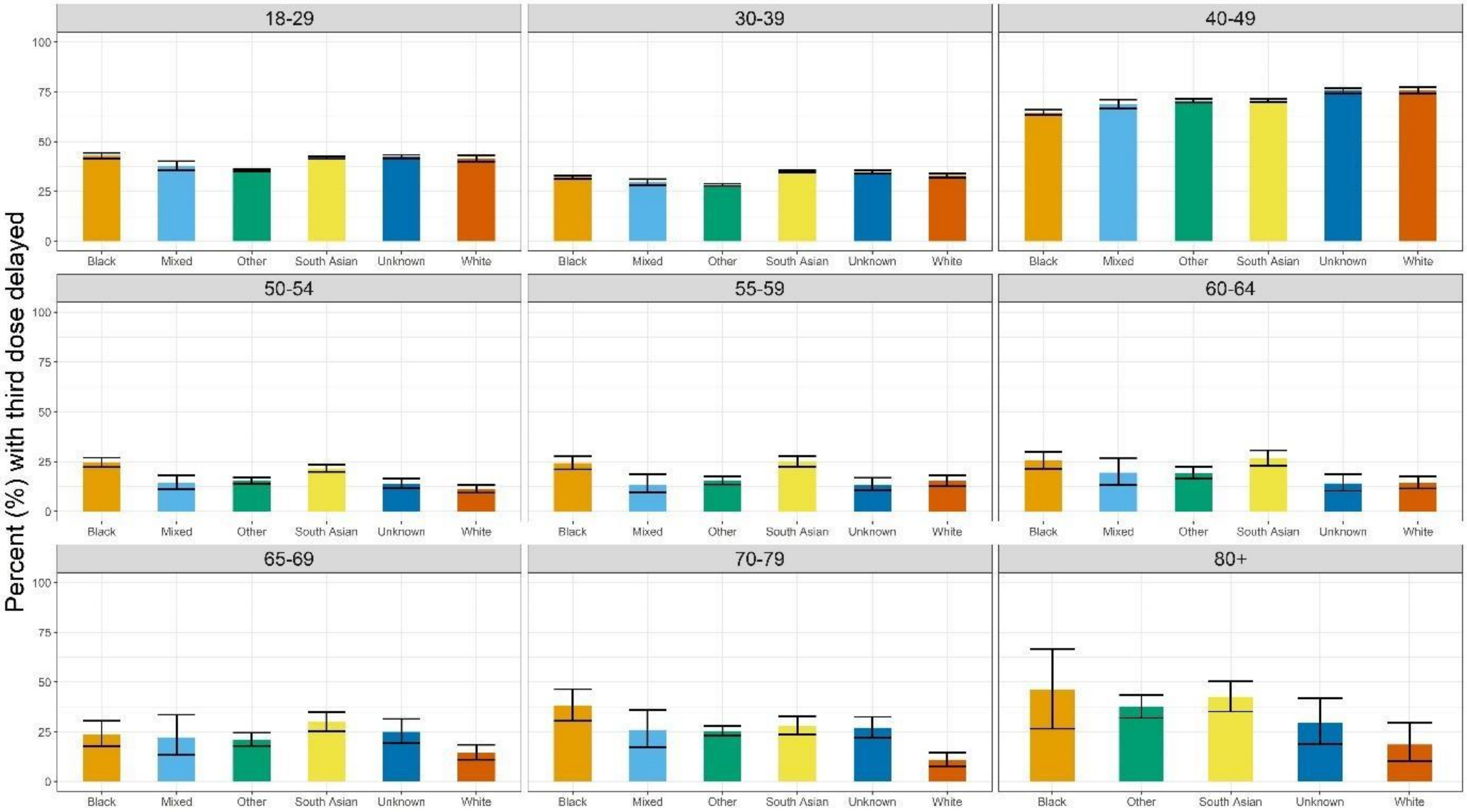

Supplementary Figure 6. Third dose delayed by ethnicity and age.
Proportion (%) and error bars represent 95% Confidence Intervals of third dose delayed by age group comparing ethnicity as of 20 April 2022

Appendix I. Results - Overdue vaccination

Supplementary Table 9. Sample size for overdue second and third COVID-19 dose vaccinations for migrants and the general population in England (as seen in Figure 2 in the main text).

Population	Age group	Second overdue Sample size (N)	Third overdue Sample Size (N)
**Million Migrant**	18-29	148795	159899
	30-39	177465	204899
	40-49	64301	69767
	50-54	10766	11410
	55-59	5620	5881
	60-64	3327	3452
	65-69	2310	2358
	70-79	3231	3271
	80+	841	845
**England**	18-29	2469782	2186149
	30-39	2446045	2269631
	40-49	2435545	2340569
	50-54	1401463	1367744
	55-59	1396906	1369893
	60-64	1210860	1191316
	65-69	1024604	1011108
	70-79	2009630	1992081
	80+	1021538	1012928

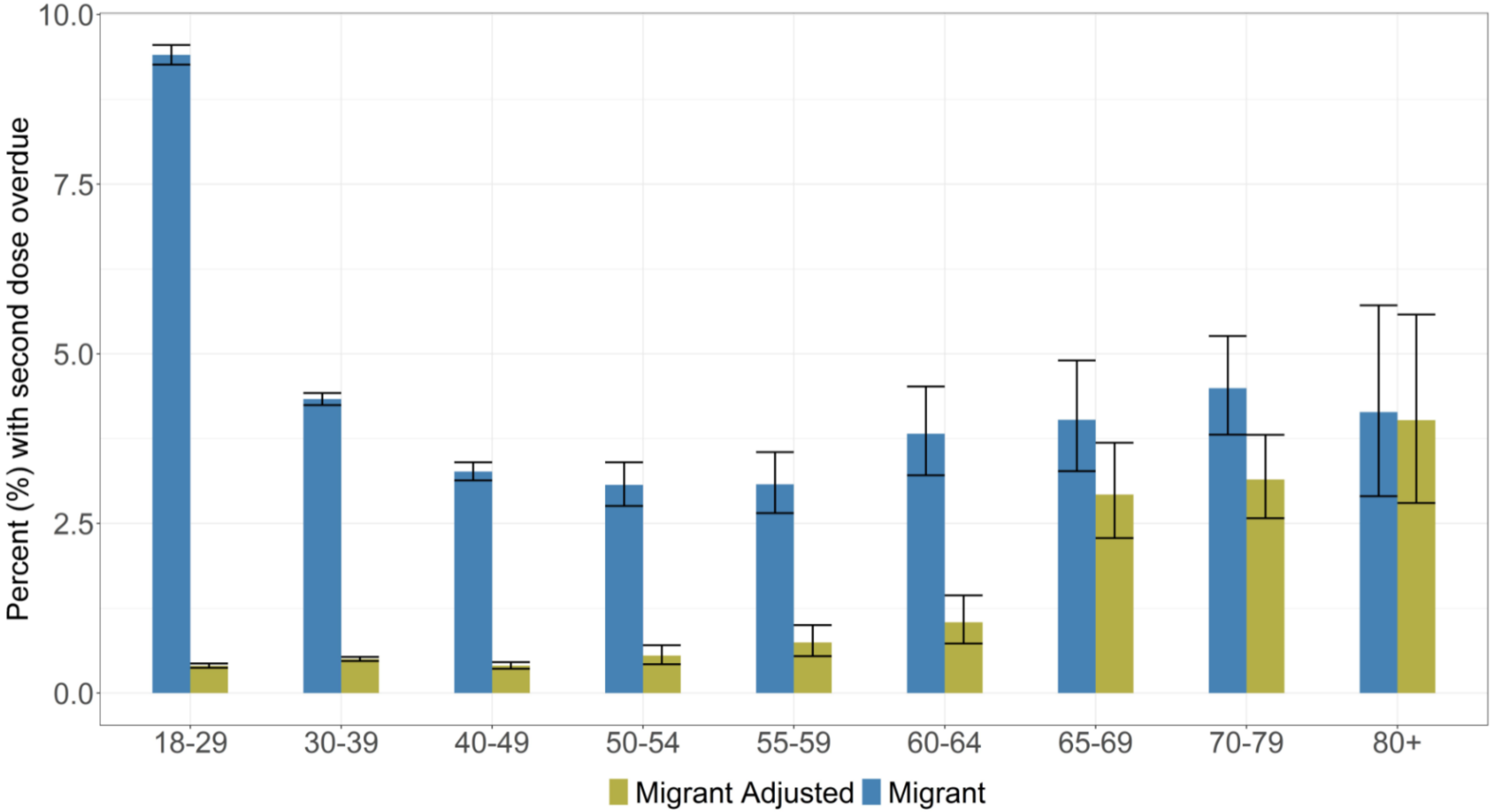

Supplementary Figure 7. Sensitivity analysis 1 - Second dose overdue for Migrant and Migrant Adjusted.
Proportion (%) and error bars represent 95% Confidence Intervals with second dose overdue comparing a shortened study end date of 01 June 2021 (Migrant Adjusted) and the original study end date of 20 April 2022 (Migrant).

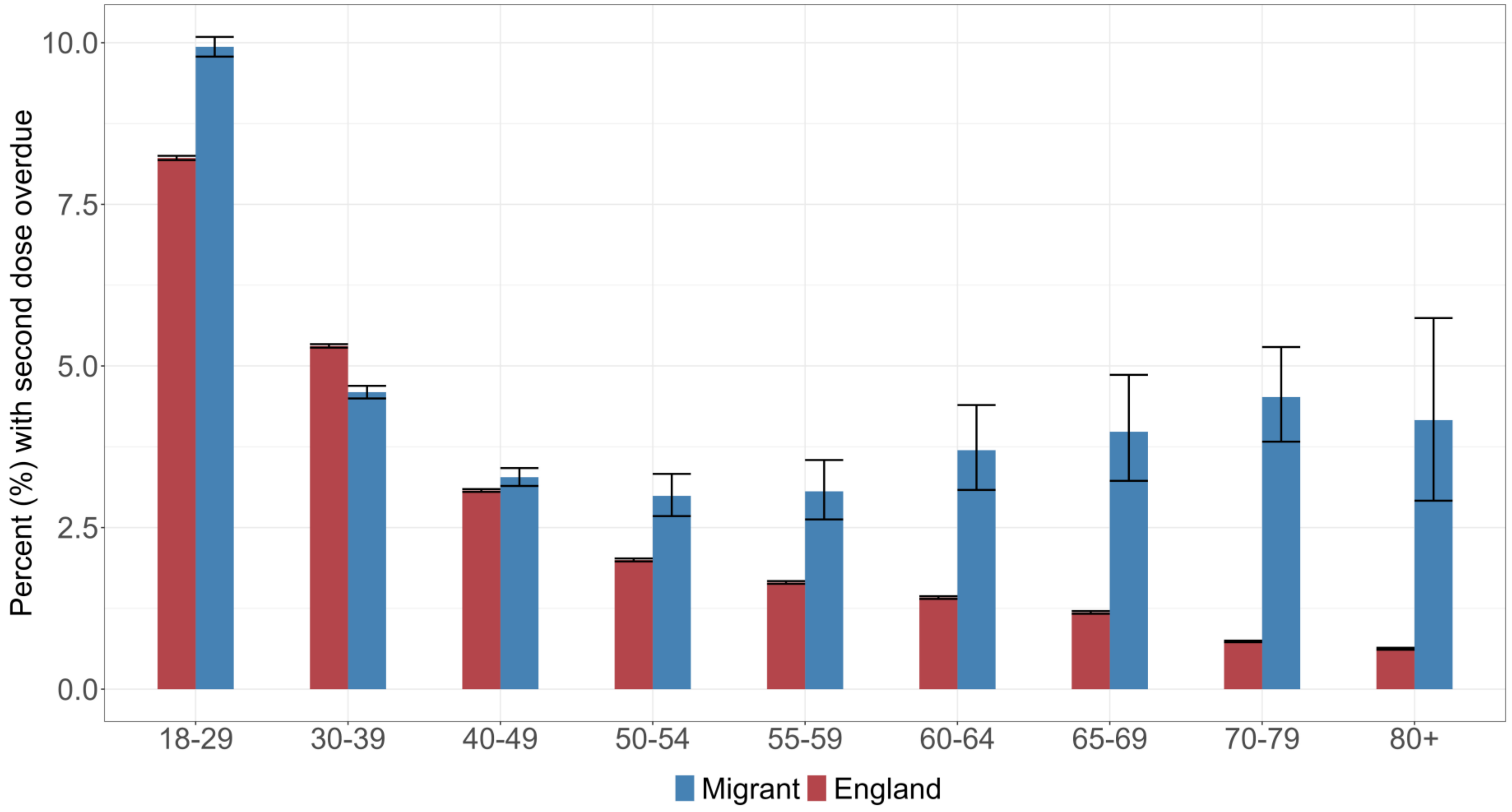

Supplementary Figure 8. Sensitivity analysis 1 – Second dose overdue for more recent migrants and England.
Proportion (%) and error bars represent 95% Confidence Intervals with second dose overdue with individuals arriving on student, work, and working holiday visas in the last five years (after 2017) within the Million Migrant-NIMS cohort (blue) to the England cohort (red) as of 20 April 2022.

Supplementary Table 10. Sample size for overdue second COVID-19 dose vaccinations by visa type and age (as seen in Figure 3 in the main text).

Age group	Visa type	Second overdue Sample size (N)
18-29	Family	7636
	Other	28179
	Refugee	2338
	Settlement	48919
	Students	59455
	Work	12378
	England	2469782
30-39	Family	13604
	Other	7409
	Refugee	2615
	Settlement	97684
	Students	42397
	Work	40060
	England	2446045
40-49	Family	4237
	Other	3505
	Refugee	1730
	Settlement	38919
	Students	9126
	Work	11714
	England	2435545
50-54	Family	748
	Other	999
	Refugee	493
	Settlement	6715
	Students	649
	Work	1679
	England	1401463
55-59	Family	368
	Other	580
	Refugee	345
	Settlement	3606
	Work	732
	England	1396906
60-64	Family	188
	Other	348
	Settlement	2181
	Work	363
	England	1210860
65-69	Other	223
	Settlement	1591
	England	1024604

Age group	Visa type	Second overdue<br>Sample size (N)
70-79	Family	193
	Other	180
	Settlement	2658
	England	2009630
80+	Settlement	714
	England	1021538

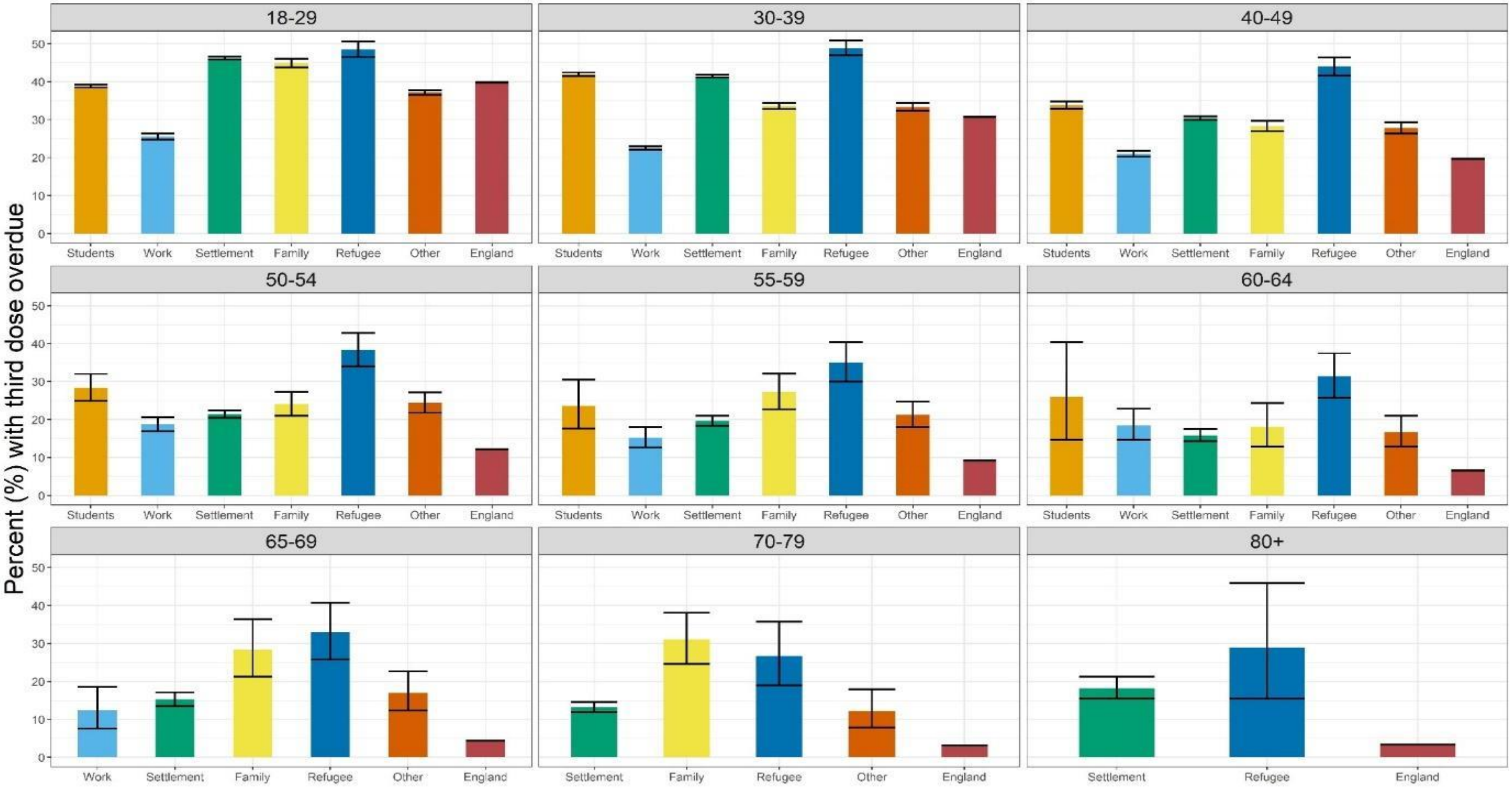

Supplementary Figure 9. Third dose overdue by visa type and age.
Proportion (%) and error bars represent 95% Confidence Intervals with third dose overdue by age group comparing visa type and England cohort as of 20 April 2022.

Supplementary Table 11. Sample size for overdue second COVID-19 dose vaccinations by ethnicity and age (as seen in Figure 4 in the main text).

Ethnicity	Age	England Sample size (N)	Migrant Sample Size (N)
Black	18-29	52556	17482
	30-39	57071	23559
	40-49	63616	10940
	50-54	27874	1980
	55-59	24101	1015
	60-64	15365	581
	65-69	8904	280
	70-79	11578	-
	80+	8463	-
Mixed	18-29	46557	4324
	30-39	38983	5876
	40-49	31059	2709
	50-54	12404	-
	55-59	10402	-
	60-64	6923	-
	65-69	4333	-
	70-79	5733	-
	80+	2730	-
Other	18-29	74144	48596
	30-39	72765	41956
	40-49	65016	15209
	50-54	23688	3021
	55-59	18606	1577
	60-64	14000	886
	65-69	10360	772
	70-79	13685	1527
	80+	5656	355
South Asian	18-29	214641	45838
	30-39	245721	98039
	40-49	220269	28366
	50-54	78183	3196
	55-59	55510	1467
	60-64	48846	749
	65-69	41202	485
	70-79	51744	547
	80+	24857	-
Unknown	18-29	335356	36859
	30-39	188839	25497
	40-49	186375	7894
	50-54	114527	1308
	55-59	106820	700
	60-64	88102	419

**Ethnicity**	**Age**	**England<br>Sample size (N)**	**Migrant<br>Sample Size (N)**
	65-69	63525	291
	70-79	93107	389
	80+	36582	-
White	18-29	1746325	6800
	30-39	1842589	9972
	40-49	1869168	4649
	50-54	1144773	1342
	55-59	1181453	816
	60-64	1037617	623
	65-69	896280	-
	70-79	1833776	413
	80+	943250	-

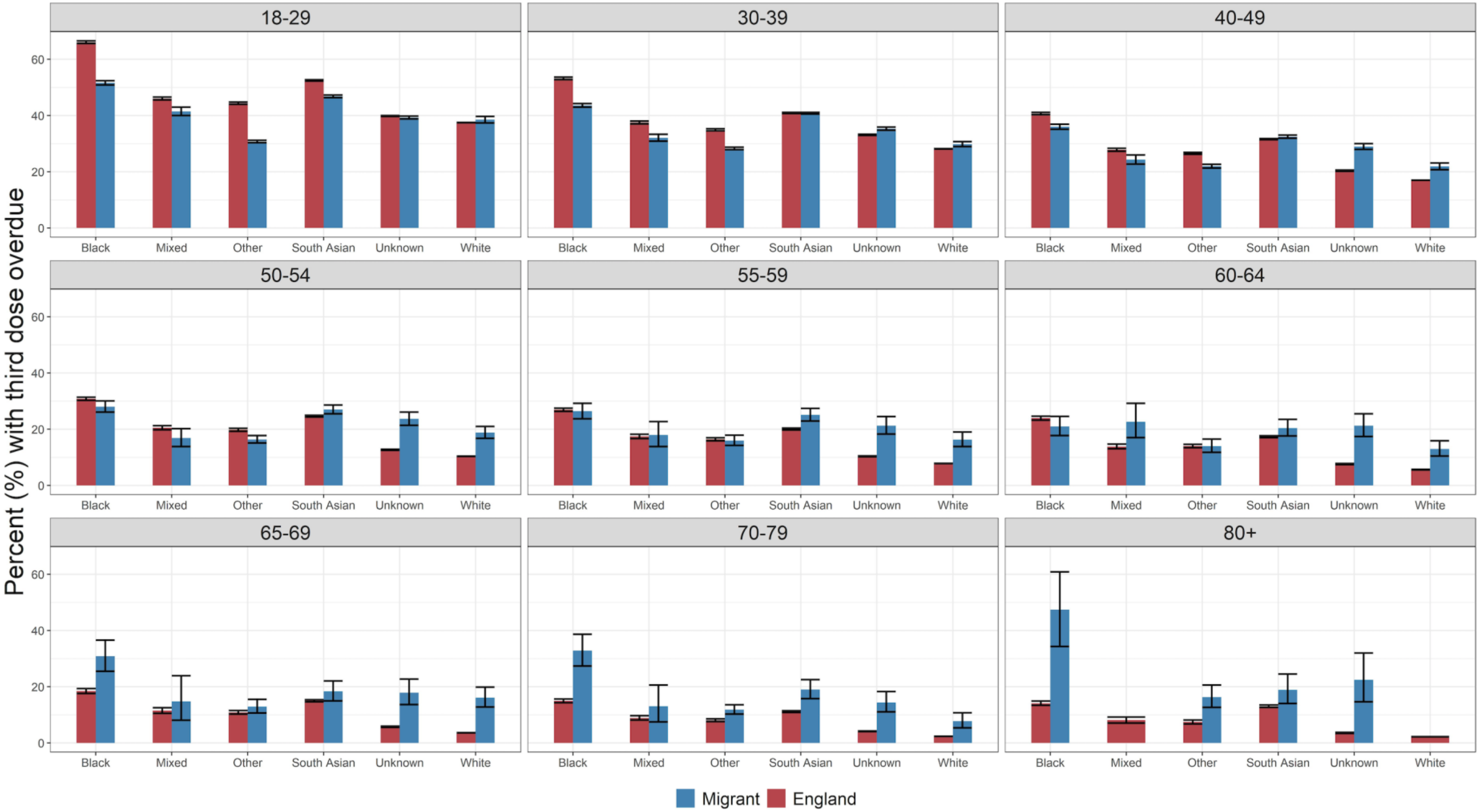

Supplementary Figure 10. **Third dose overdue by ethnicity and age.**

Proportion (%) and error bars represent 95% Confidence Intervals with third dose overdue by age group comparing ethnicity and England cohort as of 20 April 2022.

References

1 The rollout of the COVID-19 vaccination programme in England. National Audit Office, 2022 https://www.nao.org.uk/wp-content/uploads/2022/02/The-rollout-of-the-COVID-19-vaccination-programme-in-England.pdf.

2 Regulations making COVID-19 vaccination a condition of deployment to end. UK Gov. https://www.gov.uk/government/news/regulations-making-covid-19-vaccination-a-condition-of-deployment-to-end.

3 Tuberculosis tests for visa applicants. https://www.gov.uk/tb-test-visa#:~:text=You%27ll%20need%20to%20have,coughed%20up%20from%20your%20lungs.

4 Crawshaw AF, Pareek M, Were J, *et al.* Infectious disease testing of UK-bound refugees: a population-based, cross-sectional study. *BMC Med* 2018; **16**: 1–12.

5 Health Protocol: Pre-entry health assessment for UK bound refugees. Home Office, Public Health England, International Organization for Migration. 2020 June. Available from: Home Off. Public Health Engl. Int. Organ. Migr. 2020; published online June. https://assets.publishing.service.gov.uk/government/uploads/system/uploads/attachment_data/file/892799/Badged_HA_protocol_IOM_HO_PHE_-_June_2020.odt.pdf.

6 The OpenSAFELY Collaborative. OpenSAFELY Vaccine Coverage Reports. 2022 reports.opensafely.org/reports/vaccine-coverage-index.

7 Atherton F, McBride M, Smith G, Whitty C, Van-Tam J. Correspondence: Letter to the profession from the UK Chief Medical Officers regarding the UK COVID-19 vaccination programmes. 2020. https://www.gov.uk/government/publications/letter-to-the-profession-from-the-uk-chief-medical-officers-on-the-uk-covid-19-vaccination-programmes/letter-to-the-profession-from-the-uk-chief-medical-officers-regarding-the-uk-covid-19-vaccination-programmes.

8 NHS England News. NHS booster bookings open to every eligible adult. 2021. https://www.england.nhs.uk/2021/12/nhs-booster-bookings-open-to-every-eligible-adult/.

9 NHS England News. NHS begins COVID-19 booster vaccination campaign. 2021. https://www.england.nhs.uk/2021/09/nhs-begins-covid-19-booster-vaccination-campaign/.

10 NHS England News. Top up jab bookings to open up for more than one million people. 2021. https://www.england.nhs.uk/2021/11/top-up-jab-bookings-to-open-up-for-more-than-one-million-people/.

11 JCVI advice on COVID-19 booster vaccines for those aged 18 to 39 and a second dose for ages 12 to 15. UK Health Security Agency, 2021 https://www.gov.uk/government/news/jcvi-advice-on-covid-19-booster-vaccines-for-those-aged-18-to-39-and-a-second-dose-for-ages-12-to-15.
