# Supplementary material for: COVID-19 vaccination coverage for half a million non-EU migrants and refugees in England
Source: Nat Hum Behav. 2023 Dec 4;8(2):288–99. doi: 10.1038/s41562-023-01768-6 (PMC10896718; doi:10.1038/s41562-023-01768-6)
Supplement: Supplementary file 2 — Reporting Summary [file 41562_2023_1768_MOESM2_ESM.pdf]

nature portfolio

Corresponding author(s): Rachel Burns

Last updated by author(s): Oct 19, 2023

Reporting Summary

Nature Portfolio wishes to improve the reproducibility of the work that we publish. This form provides structure for consistency and transparency in reporting. For further information on Nature Portfolio policies, see our Editorial Policies and the Editorial Policy Checklist.

Statistics

For all statistical analyses, confirm that the following items are present in the figure legend, table legend, main text, or Methods section.

n/a	Confirmed	
☐	☒	The exact sample size (*n*) for each experimental group/condition, given as a discrete number and unit of measurement
☒	☐	A statement on whether measurements were taken from distinct samples or whether the same sample was measured repeatedly
☐	☒	The statistical test(s) used AND whether they are one- or two-sided<br>*Only common tests should be described solely by name; describe more complex techniques in the Methods section.*
☐	☒	A description of all covariates tested
☐	☒	A description of any assumptions or corrections, such as tests of normality and adjustment for multiple comparisons
☐	☒	A full description of the statistical parameters including central tendency (e.g. means) or other basic estimates (e.g. regression coefficient) AND variation (e.g. standard deviation) or associated estimates of uncertainty (e.g. confidence intervals)
☐	☒	For null hypothesis testing, the test statistic (e.g. *F*, *t*, *r*) with confidence intervals, effect sizes, degrees of freedom and *P* value noted<br>*Give P values as exact values whenever suitable.*
☒	☐	For Bayesian analysis, information on the choice of priors and Markov chain Monte Carlo settings
☒	☐	For hierarchical and complex designs, identification of the appropriate level for tests and full reporting of outcomes
☒	☐	Estimates of effect sizes (e.g. Cohen's *d*, Pearson's *r*), indicating how they were calculated

Our web collection on statistics for biologists contains articles on many of the points above.

Software and code

Policy information about availability of computer code

Data collection	EMIS or TPP software used by OpenSAFELY to identify GP patients.
Data analysis	R Studio version 4.1.2 was used in the analysis. Code available: https://doi.org/10.5281/zenodo.8095568

For manuscripts utilizing custom algorithms or software that are central to the research but not yet described in published literature, software must be made available to editors and reviewers. We strongly encourage code deposition in a community repository (e.g. GitHub). See the Nature Portfolio guidelines for submitting code & software for further information.

Data

Policy information about availability of data

All manuscripts must include a data availability statement. This statement should provide the following information, where applicable:

- Accession codes, unique identifiers, or web links for publicly available datasets
- A description of any restrictions on data availability
- For clinical datasets or third party data, please ensure that the statement adheres to our policy

The data that support the findings of this study are held by UKHSA and OHID within the UK government. Access to the data is currently restricted and not publicly available. Researchers interested in working with these datasets should contact these agencies directly. The authors (RB) will apply for approval to create an anonymised version of the data by 2024 available upon request for research and replication purposes, provided that the request come from individuals affiliated

with a university. If approved, the data will be held in the UCL Data Safe Haven and will be maintained until 2034. OpenSAFELY dataset is publicly available: https://reports.opensafely.org/reports/vaccine-coverage-index/.

Research involving human participants, their data, or biological material

Policy information about studies with human participants or human data. See also policy information about sex, gender (identity/presentation), and sexual orientation and race, ethnicity and racism.

Reporting on sex and gender	Sex was collected and overall proportions of the outcomes are disaggregated by sex.
Reporting on race, ethnicity, or other socially relevant groupings	Ethnicity was collected and overall proportions of the outcomes are disaggregated by ethnicity.
Population characteristics	We used the following variables to compare the Million Migrant-NIMS cohort with the England cohort: age, visa type, and ethnicity. Age in years was aggregated to match OpenSAFELY age groups: 16-17, 18-29, 30-39, 40-49, 50-54, 55-59, 60-64, 65-69, 70-79, and 80+. Visa type consisted of seven categories: Family Reunion, Settlement and Dependents, Student, Refugee, Work, Other, and England cohort (i.e., individuals in England comparison population). Ethnicity was aggregated according to ONS census 2011 categories: White, South Asian, Black, Mixed, Other and Unknown. Ethnicity data were only available for people aged >=18 in the England cohort. We also selected the following variables to display using descriptive statistics given their association with migration and vaccination uptake: sex, region of origin, year of arrival to England, and region resident in England. No information on death, contraindications, or emigration out of England were available. Variable definitions and further description of confounder selection for the final statistical models are available in Appendix D and E.
Recruitment	No recruitment as this was a data linkage population-based cohort study using administrative data.
Ethics oversight	United Kingdom Health Security Agency; NHS England Ethics, UCL Ethics

Note that full information on the approval of the study protocol must also be provided in the manuscript.

Field-specific reporting

Please select the one below that is the best fit for your research. If you are not sure, read the appropriate sections before making your selection.

☐ Life sciences ☒ Behavioural & social sciences ☐ Ecological, evolutionary & environmental sciences

For a reference copy of the document with all sections, see nature.com/documents/nr-reporting-summary-flat.pdf

Behavioural & social sciences study design

All studies must disclose on these points even when the disclosure is negative.

Study description	This was a quantitative study. We conducted a retrospective population-based cohort study using the Million Migrant cohort linked to England's National Immunisation Management Service (NIMS) which acts as a national vaccine register for COVID-19 vaccinations. The Million Migrant-NIMS cohort was compared to England's general population using the publicly available OpenSAFELY dataset.18 Our study period began on 8 December 2020, the start of the staggered roll out of the UK national COVID-19 vaccination campaign, and ended 20 April 2022.
Research sample	The Million Migrant-NIMS cohort<br>The Million Migrant cohort consists of two data sources: first, the non-EU migrant pre-entry tuberculosis screening dataset collected as part of the UK visa application process and second, the refugee pre-arrival health assessment dataset collected for all refugees enrolled in a UK refugee resettlement programme (details in Appendix B). The most recent record for individuals aged >=16 was deterministically linked by forename, surname, date of birth, and sex to the NHS Personal Demographic Service (PDS) by NHS Digital's Demographic Batch Service to obtain an individual's unique patient identifier, National Health Service (NHS) number, and UK postcode. The Million Migrant cohort (supplemented with NHS numbers from PDS) was deterministically linked by NHS number, date of birth, sex, and where available UK postcode to the NIMS COVID-19 vaccination dataset held at the United Kingdom Health Security Agency (UKHSA). The linkage followed a stepwise deterministic matching procedure adapted from methodology used by NHS Digital (details on linkage methodology in Appendix C). This cohort is representative of non-EU migrants who had a pre-entry TB screening and refugees who had a pre-arrival health assessment. These data were available for the author (RB) and represent a unique opportunity to study migrant.<br><br>The England cohort<br>To provide a representative comparison population for England (referred to as the England cohort), we used publicly available aggregate data from the OpenSAFELY cohort. The OpenSAFELY cohort included all patients registered with a general practice using either EMIS or TPP software in England. Information on vaccination status was linked back to the participants' primary care records following their vaccination. Vaccination status and date of vaccination was ascertained by the presence of any recorded COVID-19 vaccine administration code in their primary care record. Total aggregate second and third dose data were available for people aged >=18 (further details on study data sources in Appendix D). This cohort has been found to be representative of the entire population in England.

Sampling strategy	No sampling strategy was used in the Million Migrant cohort as this is a data linkage cohort study linking existing datasets. The population-based cohort is highly powered and no sampling was conducted.<br><br>OpenSAFELY: Primary care records managed by the GP software providers EMIS and TPP were accessed through OpenSAFELY, an open-source data analytics platform created by the author team on behalf of NHS England to address urgent COVID-19 research questions (https://opensafely.org). OpenSAFELY provides a secure software interface allowing a federated analysis of pseudonymised primary care patient records from England in near real-time within the EMIS and TPP highly secure data environments. Nondisclosive, aggregated results are exported to GitHub where further data processing and analysis takes place. For the descriptive analysis all patients registered with a general practice using EMIS and TPP were included. As a result, there was no sampling strategy used in this dataset.
Data collection	We did not collect any data in this study, as a result the researchers did not need to be blind. We linked data from non-EU migrants and resettled refugees to the national COVID-19 vaccination dataset in England.<br><br>The non-EU migrant data (names, date of birth, sex) was collected by clinical staff outside of the UK as part of the pre-entry TB screening programmes. The refugee data (names, date of birth, sex) was collected by clinical staff outside of the UK as part of the pre-arrival health assessment programmes. Personal Demographic Service data is collected at any NHS service point (hospital, GP) and the COVID-19 vaccination data was collected at an vaccination point in the UK or recorded if received the vaccination abroad. The researchers did not collect any data.
Timing	Our study period began on 8 December 2020, the start of the staggered roll out of the UK national COVID-19 vaccination campaign, and ended on 20 April 2022.
Data exclusions	No data exclusions.
Non-participation	No participants were included in the study as this study used administrative data.
Randomization	Randomization was not conducted. This study used administrative data to conduct a cohort study without groups of participants. Descriptive analysis was conducted based on demographic groups.

Reporting for specific materials, systems and methods

We require information from authors about some types of materials, experimental systems and methods used in many studies. Here, indicate whether each material, system or method listed is relevant to your study. If you are not sure if a list item applies to your research, read the appropriate section before selecting a response.

Materials & experimental systems

n/a	Involved in the study	
☒	☐	Antibodies
☒	☐	Eukaryotic cell lines
☒	☐	Palaeontology and archaeology
☒	☐	Animals and other organisms
☒	☐	Clinical data
☒	☐	Dual use research of concern
☒	☐	Plants

Methods

n/a	Involved in the study	
☒	☐	ChIP-seq
☒	☐	Flow cytometry
☒	☐	MRI-based neuroimaging

Plants

Seed stocks	*Report on the source of all seed stocks or other plant material used. If applicable, state the seed stock centre and catalogue number. If plant specimens were collected from the field, describe the collection location, date and sampling procedures.*
Novel plant genotypes	*Describe the methods by which all novel plant genotypes were produced. This includes those generated by transgenic approaches, gene editing, chemical/radiation-based mutagenesis and hybridization. For transgenic lines, describe the transformation method, the number of independent lines analyzed and the generation upon which experiments were performed. For gene-edited lines, describe the editor used, the endogenous sequence targeted for editing, the targeting guide RNA sequence (if applicable) and how the editor was applied.*
Authentication	*Describe any authentication procedures for each seed stock used or novel genotype generated. Describe any experiments used to assess the effect of a mutation and, where applicable, how potential secondary effects (e.g. second site T-DNA insertions, mosiacism, off-target gene editing) were examined.*
